# Exploration of CviR-mediated quorum sensing inhibitors from *Cladosporium* spp. against *Chromobacterium violaceum* through computational studies

**DOI:** 10.1038/s41598-023-42833-4

**Published:** 2023-09-19

**Authors:** Mahadevamurthy Murali, Faiyaz Ahmed, Hittanahallikoppal Gajendramurthy Gowtham, Jamiu Olaseni Aribisala, Rukayat Abiola Abdulsalam, Ali A. Shati, Mohammad Y. Alfaifi, R. Z. Sayyed, Saheed Sabiu, Kestur Nagaraj Amruthesh

**Affiliations:** 1 Applied Plant Pathology Laboratory, Department of Studies in Botany, University of Mysore, Manasagangotri, Mysore, 570006 India; 2 Department of Clinical Nutrition, College of Applied Health Sciences in Ar Rass, Qassim University, 51452 Buraydah, Saudi Arabia; 3 Department of PG Studies in Biotechnology, Nrupathunga University, Nrupathunga Road, Bangalore, 560001 India; 4 Department of Biotechnology and Food Science, Faculty of Applied Sciences, Durban University of Technology, Durban, South Africa; 5 Faculty of Science, Biology Department, King Khalid University, 9004 Abha, Saudi Arabia; 6 Department of Microbiology, PSGVP Mandal’s S I Patil Arts, G B Patel Science and STKV Sangh Commerce College, Shahada, 425409 India

**Keywords:** Drug discovery, Plant sciences, Diseases

## Abstract

An opportunistic human pathogenic bacterium, *Chromobacterium violaceum* resists the potency of most antibiotics by exploiting the quorum sensing system within their community to control virulence factor expression. Therefore, blocking the quorum sensing mechanism could help to treat several infectious caused by this organism. The quorum sensing receptor (CviR) of *C. violaceum* was used as a model target in the current investigation to identify potentially novel quorum sensing inhibitors from *Cladosporium* spp. through in silico computational approaches. The molecular docking results confirmed the anti-quorum sensing potential of bioactive compounds from *Cladosporium* spp. through binding to CviR with varying docking scores between – 5.2 and – 9.5 kcal/mol. Relative to the positive control [Azithromycin (– 7.4 kcal/mol)], the top six metabolites of *Cladosporium* spp. had higher docking scores and were generally greater than – 8.5 kcal/mol. The thermodynamic stability and binding affinity refinement of top-ranked CviR inhibitors were further studied through a 160 ns molecular dynamic (MD) simulation. The Post-MD simulation analysis confirmed the top-ranked compounds' affinity, stability, and biomolecular interactions with CviR at 50 ns, 100 ns, and 160 ns with Coniochaetone K of the *Cladosporium* spp. having the highest binding free energy (– 30.87 kcal/mol) and best interactions (two consistent hydrogen bond contact) following the 160 ns simulation. The predicted pharmacokinetics properties of top selected compounds point to their drug likeliness, potentiating their chance as a possible drug candidate. Overall, the top-ranked compounds from *Cladosporium* spp., especially Coniochaetone K, could be identified as potential *C. violaceum* CviR inhibitors. The development of these compounds as broad-spectrum antibacterial medicines is thus possible in the future following the completion of further preclinical and clinical research.

## Introduction

*Chromobacterium violaceum* is an opportunistic pathogen that commonly causes infections such as bacteremia and visceral abscesses in humans^1^, which require effective treatment measures to control potentially life-threatening diseases. Because of the organism's ability to resist the effects of most common antibiotics, several research efforts are focused on discovering and developing a more effective strategy to combat the infections caused by the organism. One of these strategies is inhibiting their quorum-sensing phenomenon, a cell-to-cell communication mediated by small autoinducing signaling molecules that allow bacteria to respond and adapt according to the density of their cell population by altering their gene expression. The quorum-sensing systems play an essential role in determining how *C. violaceum* expresses virulence factors that support its colonization, adhesion, invasion, and toxin production, thus causing infections that are relatively difficult to treat^2–4^. Therefore, targeting quorum-sensing systems could help stop the pathogenesis of *C. violaceum* and prevent the development and progression of life-threatening diseases that may arise due to *C. violaceum* pathogenesis.

In *C. violaceum*, the LuxI/LuxR and CviI/CviR systems control virulence factor expression and the development of other phenotypic traits, thus activating the quorum-sensing system^5^. The CviR codes for the DNA binding cytoplasmic transcription factor, acting as the primary quorum regulator in *C. violaceum*, which could serve as an ideal quorum-sensing target protein^6^. Several studies have demonstrated the capability of plants' secondary metabolites to inhibit the quorum-sensing system of *C. violaceum*^5,7,8^. Rasoanaivo et al.^7^ showed that the crude extracts of several plant parts are effective in inhibiting *C. violaceum* quorum sensing compared to their purified components. The effectiveness of crude extracts is attributed to the synergistic interactions between the different compounds of the crude extract to their components. Hence, it has been suggested that crude extracts might be the best method for blocking quorum sensing of several multidrug-resistant bacteria rather than their pure compound. From the literature, it is well documented that the species of *Cladosporium* possesses a rich source of diverse bioactive compounds which are used as antimicrobial, anticancer, antiviral, etc.^9,10^. With the available information on the anti-quorum sensing potential of plant secondary metabolites, little or no information about the antagonistic effects of microbial metabolites are known, even though the microbial communities harbor several extracellular small anti-quorum sensing signaling molecules. Therefore, the present study focused on identifying the potential CviR-mediated quorum-sensing inhibitors from the bioactive compounds of *Cladosporium* spp. against *C. violaceum* through computational methods. To our knowledge, this is the new attempt to explore the bioactive compounds from microbial source (viz., *Cladosporium* spp.) as the potential *C. violaceum* CviR-mediated quorum sensing inhibitors.

## Materials and methods

### Metabolite profiling and identification

The ligand molecules selected in the current investigation concerned the three-dimensional (3D) structure of 123 bioactive compounds from *Cladosporium* spp.^9^. Azithromycin was used as a positive control since it is commonly used for treating various bacterial infections^11^. The 3D structure of bioactive compounds documented by Salvatore et al.^9^ were retrieved from the PubChem compound database in structured data format (SDF) for molecular docking. ChemAxon's Marvin JS chemical drawing tool version 18.30 was used to draw those unavailable in the database. The OpenBabel version 2.3.2 was employed to convert the chemical data from SDF to protein data bank (PDB) format^12^. Before the molecular docking study, the PRODRG server was used to optimize the geometry of ligand PDB files.

### Protein target identification, molecular docking, and molecular dynamic simulation

Research Collaboratory for Structural Bioinformatics Protein Data Bank (RCSB PDB) database was used to retrieve the 3D X-Ray crystallographic structure of the protein in PDB format. Based on the literature, the crystal structure of the CviR receptor from *C. violaceum* 12,472 bound to C6-HSL with PDB ID: 3QP6^13^ was selected as a model target protein in the present study. Before the molecular docking experiment, the protein structure was processed by deleting the co-crystallized water, ligand molecules attached, and hydrogen atoms and Kollmann charges. Then, the processed structure was energy minimized with empirical force fields using Swiss-Pdb Viewer software (version 4.10) to get a lower energy conformation, which denotes a more stable conformation. The process for modeling the protein structure effectively optimized the conformational errors in the structural geometry. The steepest descent algorithm with GROMOS96 force field was also used in the geometry optimization process^14^.

The Ramachandran plot, which infers the reliability of the SWISS-MODEL server-developed protein structure model, was used to validate further the CviR protein's model structure in the PROCHECK online server. The plot allows for the visualization of highly favored, allowed, and disallowed φ (phi) and ψ (psi) angles of amino acid residues in a protein backbone structure. Additionally, the protein model quality was tested using the Protein Structure Analysis (ProSA) online tool, which validates the high quality of protein when its Z-score falls within the range of native protein.

The molecular docking experiment was achieved using the PyRx 0.8 program based on the Lamarckian genetic algorithm to examine how the phytocompounds bind with the active site residues of the CviR protein structure^15^. The cubical grid box that encloses the active sites of CviR had the dimension of 60 × 60 × 60 along the x, y, and z axis, respectively, with a default grid spacing value of 0.375 Å. The target protein receptor was contained within a grid box of specified dimensions with docking exhaustiveness of 100 poses. The protein receptor and ligand coordinates were then stored in a pdbqt file format with the docking settings set to default. The binding affinities, expressed in kilocalories per mole (kcal/mol), were used to depict the docking data. In molecular docking analysis, the docked conformation with the lowest binding energy was regarded as the ligand's optimal binding posture with the CviR protein. After docking, the BIOVIA Discovery Studio software was exploited to visualize ligand interaction with protein structure. The compounds used in the present study were re-docked (self-docked) with the CviR protein to standardize the docking protocol and to get the correct binding pose of ligands in the co-crystallized protein structure.

In addition, the MD simulations were performed on the six compounds with the highest binding affinity for the CviR protein compared to the positive control (Azithromycin). The MD simulations were executed using AMBER 18 software in the High-Performance Computing Centre, explained in our earlier studies^16^, where the Amber FF18SB force field variant provided the operating system functions. The atomic partial charges of ligand via general amber force fields (GAFF) and restrained electrostatic potentials (RESP) were created using the ANTECHAMBER package. The TIP3P potential represented water molecules, counter ions (such as Na^+^/Cl^–^) were added to neutralize the system, and 8 Å was chosen as the cut-off value for non-bond interactions. The total simulation was carried out at 160 ns with the Leap module SHAKE algorithm employed to constrain the expansion of all chemical bonding, including hydrogen atoms. For each simulation, the time step size of 2 fs that corresponds to an isothermal-isobaric (NPT) ensemble containing randomized seeding, 300 K temperature, 1 bar constant pressure, Langevin thermostat (1.0 ps collision frequency), and 2 ps pressure-coupling constant. The examined MD simulation results were also presented as the post-dynamic simulation data.

Further, the post-dynamic simulation was achieved, as explained in our earlier studies^16^. AMBER 14 PTRAJ program was used to combine and analyze the coordinates of the systems, followed by the CPPTRAJ program used for the analyses of parameters viz., root mean square deviation (RMSD), root mean square fluctuation (RMSF), radius of gyration (RoG), solvent accessible surface area (SASA), and number of intra-molecular hydrogen (H) bonds. The molecular Mechanics/GB Surface Area (MM/GBSA) approach was employed to calculate the average binding free energy for forming a protein–ligand complex over 100,000 snapshots from the 160 ns simulation. The Discovery Studio software version 21.1.0 was exploited to analyze and visualize the interaction of each complex. The Origin data analysis and graphing software V18 generated all the data plots^17^.

### Pharmacokinetics and toxicity evaluation

The open access in silico tools for early prediction of pharmacokinetics properties like absorption, distribution, metabolism, excretion, and toxicity (ADMET) properties of drugs has revolutionized the strategies for disease management. The target ligand molecules' physicochemical parameters and ADMET properties were predicted using the pkCSM ADMET descriptors algorithm, making it easier to find potential treatment possibilities^12,18^.

## Results and discussion

**Figure 1:**
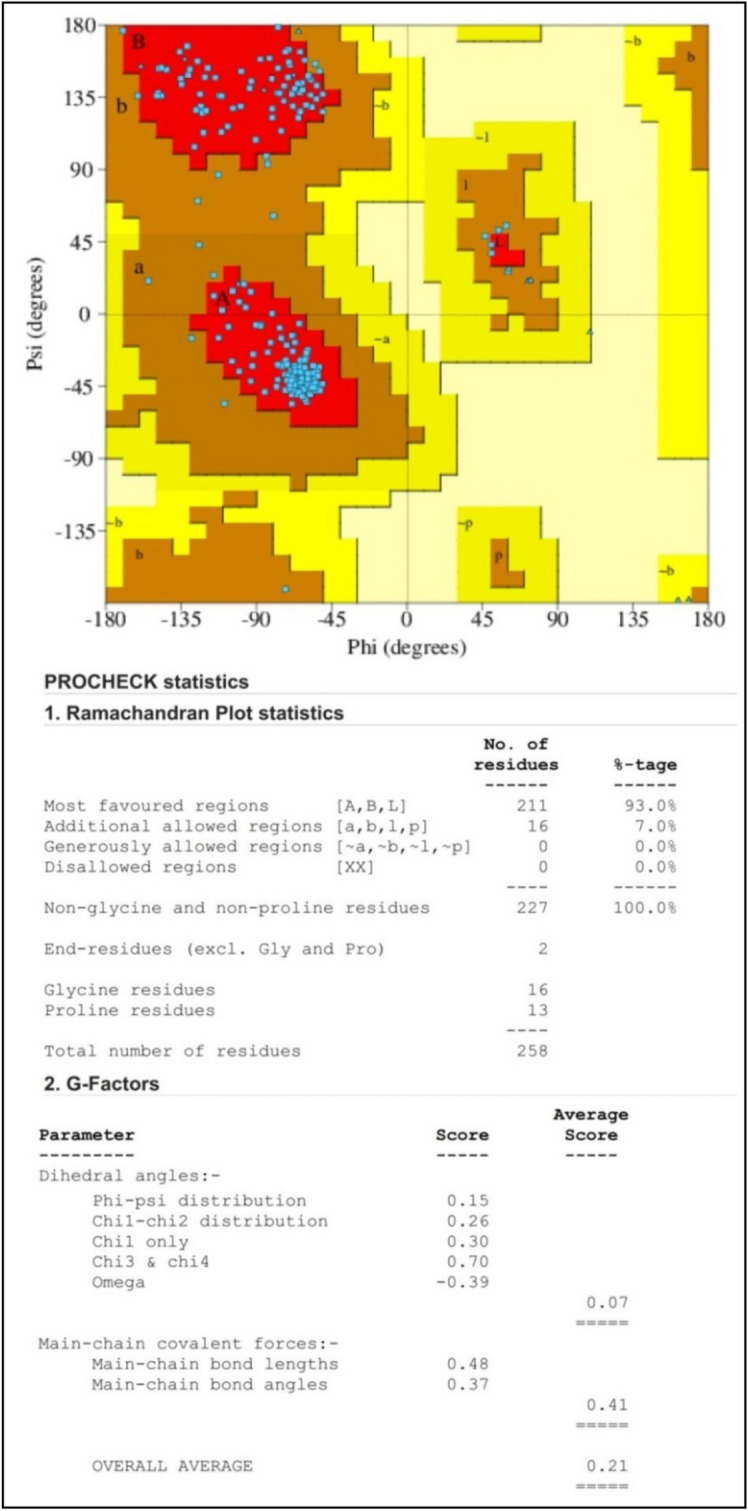
Ramachandran plot analysis of *C. violaceum* CviR protein receptor (PDB: 3QP6) structure. The graph's red, brown, and yellow regions designate the protein residues in most allowed, additionally allowed, and generously allowed regions.

**Figure 2:**
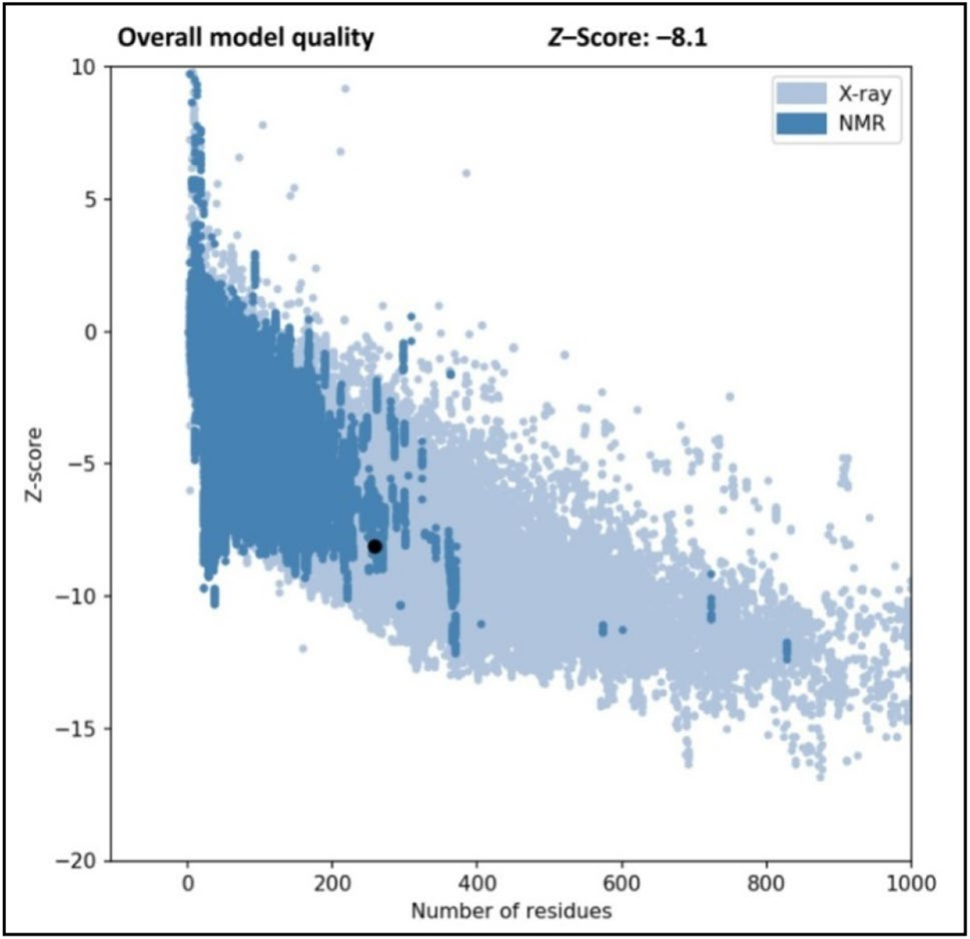
Z-score for *C. violaceum* CviR protein receptor (PDB: 3QP6) structure.

Quorum sensing has emerged as an attractive target for developing novel therapeutic drugs and reducing bacterial pathogenicity since it regulates the virulence factors in many pathogenic bacteria^2^. This study exploited computational techniques (such as molecular docking, MD simulation and pharmacokinetics) to identify potent *C. violaceum* quorum sensing inhibitors from *Cladosporium* spp. by targeting the CviR protein. The CviR protein was identified as a key drug target to reduce the virulence properties and pathogenesis of *C. violaceum*. The Ramachandran plot revealed that most amino acid residues of the CviR protein were found within the most favored regions (Fig. 1). The ProSA Z-score of –8.1 was found within the characteristic range of native protein conformation, indicating that the protein structure had extremely few errors (Fig. 2).

### Molecular docking and validation

**Figure 3:**
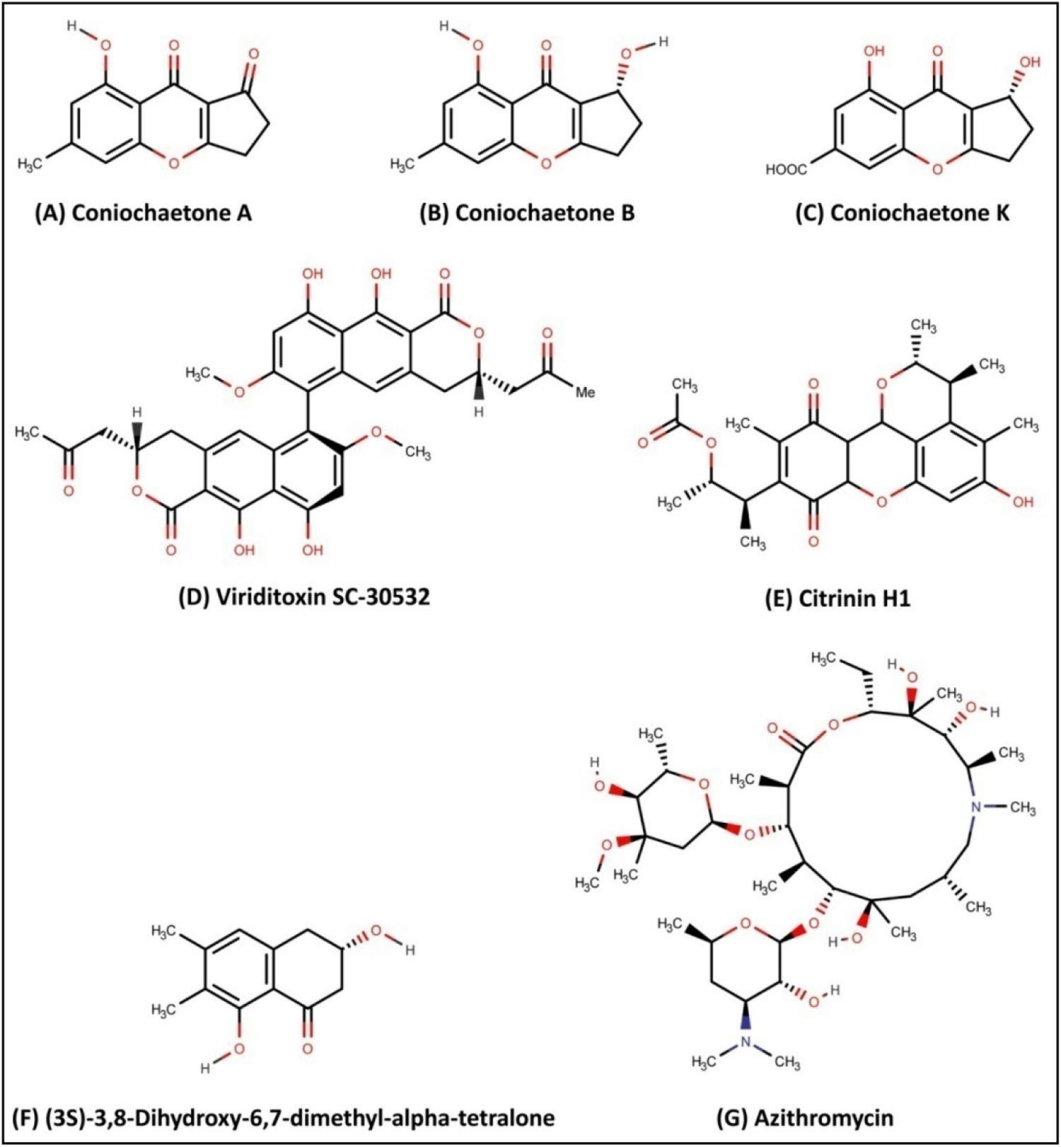
Chemical structure of the potential compounds from *Cladosporium* spp. and positive control.

**Figure 4:**
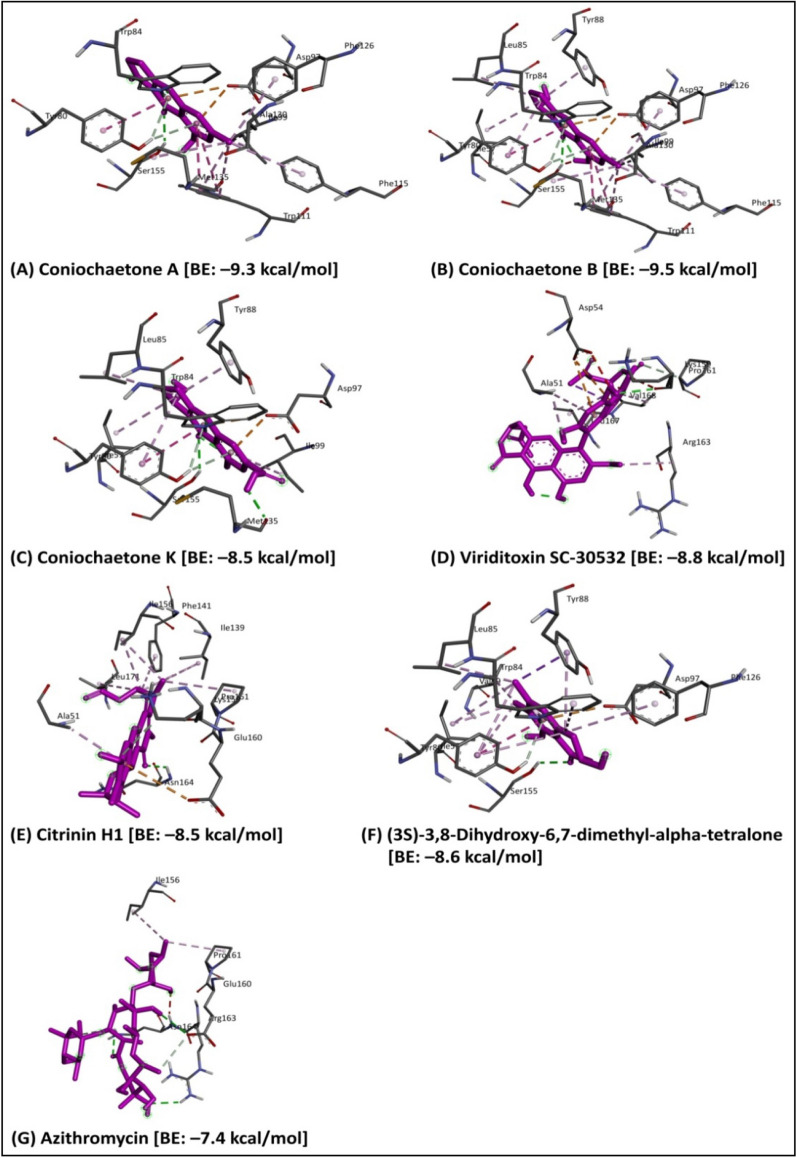
Three-dimensional illustration of the *C. violaceum* CviR protein receptor (PDB: 3QP6) interaction with the potential compounds from *Cladosporium* spp. and positive control. The hydrogen bonds and hydrophobic interaction are represented by green and purple dashed lines, respectively.

**Figure 5:**
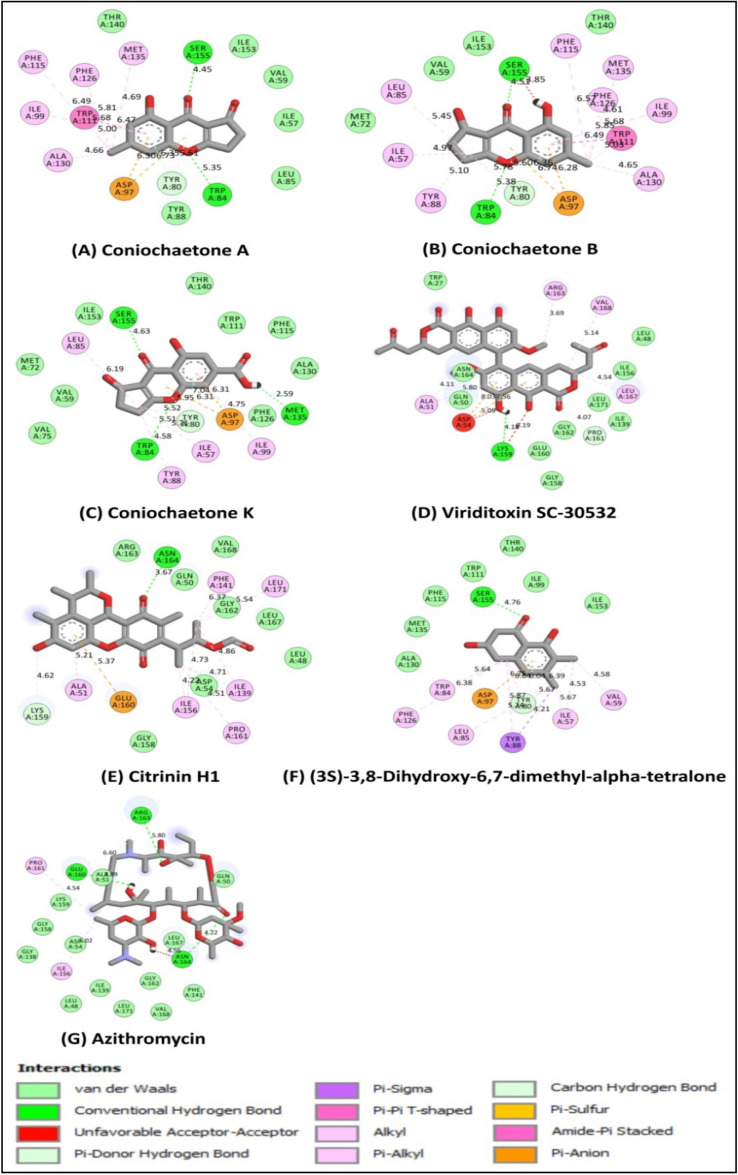
Two-dimensional illustration of the *C. violaceum* CviR protein receptor (PDB: 3QP6) interaction with the potential compounds from *Cladosporium* spp. and positive control.

Molecular docking is a powerful tool for predicting the predominant ligand binding modes with a known 3D protein structure^19^. The molecular docking of 123 bioactive compounds from *Cladosporium* spp. (Supplementary Table 1) with the CviR (PDB: 3QP6) had affinity ranging between – 5.2 and – 9.5 kcal/mol. The top six compounds which include Coniochaetone A (– 9.3 kcal/mol), Coniochaetone B (– 9.5 kcal/mol), Coniochaetone K (– 8.5 kcal/mol), Viriditoxin SC-30532 (– 8.8 kcal/mol), Citrinin H1 (– 8.5 kcal/mol), and (3S)-3,8-Dihydroxy-6,7-dimethyl-alpha-tetralone (– 8.6 kcal/mol) had higher docking score that Azithromycin (– 7.4 kcal/mol). This observation points to the top-ranked compounds' better affinity for CviR, suggesting their advantage as an active anti-quorum sensing inhibitor over Azithromycin (Table 1). Figure 3 represents the chemical structure of top-ranked compounds from *Cladosporium* spp. and positive control. The effective drug development process requires the computational analysis of protein–ligand interactions. The different types of protein–ligand interactions (such as hydrogen bonding, van der Waals force, electrostatic interaction and hydrophobic interaction) have a central role in regulating biological function, including transcription, translation and signal transduction^20^. The higher docking score of Coniochaetone B (– 9.5 kcal/mol) against CviR indicates that it is the most potent active anti-quorum sensing compound from *Cladosporium* spp. with important hydrogen bond interactions with Trp84 and Ser155 residues (Figs. 4 and 5).

**Table 1:** Molecular docking of bioactive compounds from *Cladosporium* spp. and positive control against CviR protein of *C. violaceum*.

Compound name	Binding energy (kcal/mol)
Coniochaetone A	– 9.3
Coniochaetone B	– 9.5
Coniochaetone K	– 8.5
Viriditoxin SC-30532	– 8.8
Citrinin H1	– 8.5
(3*S*)-3,8-Dihydroxy-6,7-dimethyl-alpha-tetralone	– 8.6
Azithromycin [positive control]	– 7.4

Similarly, Coniochaetone A (– 9.3 kcal/mol), with the second highest docking score, also interacted through hydrogen bond contacts with Trp84 and Ser155 of CviR, thus suggesting the importance of the residue in the binding of protein (Figs. 4 and 5). The positive control Azithromycin, however, interacted with hydrogen bond contact with Glu160, Arg163, and Asn164 residues of CviR protein (Figs. 4 and 5). The virtual screening of natural products showed that Butein and Bavachin were found to inhibit the *C. violaceum* with the least Glide score − 11.246 and − 8.056 kcal/mol, by interacting with more number of hydrogen bonds in the ligand binding domain of CviR protein^21^. Venkatramanan et al.^5^ have reported that hexadecanoic acid, 2-hydroxy-1-(hydroxymethyl) ethyl ester of *Passiflora edulis* could remarkably interact with the active site of CviR protein with a least binding energy of − 8.825 kcal/mol to inhibit the quorum sensing system in *C. violaceum*. The in silico molecular interaction of thymol showed the Glide score of − 5.847 kcal/mol through the H-bonding (SER-155) and Pi-Pi stacking (TYR-80) interactions in CviR protein^22^. Recently, Chaieb et al.^23^ have demonstrated that Catechin and Nakinadine B were found to show the best Glide scores − 9.93 and − 10.34 kcal/mol, respectively, for the interaction with CviR protein.

### Molecular dynamic simulation

The MD simulation is the most sophisticated technique for understanding the structure-to-function relationship between the diverse biological macromolecules complexed with various small molecules^24^. The MD simulation can capture the essential biomolecular processes (viz., protein folding, membrane transport, ligand binding, and protein conformational change) and predict how the biomolecules will respond to perturbations such as mutation, protonation states, phosphorylation, and addition/ removal of ligand at an atomic level^25^.

### RMSD

**Figure 6:**
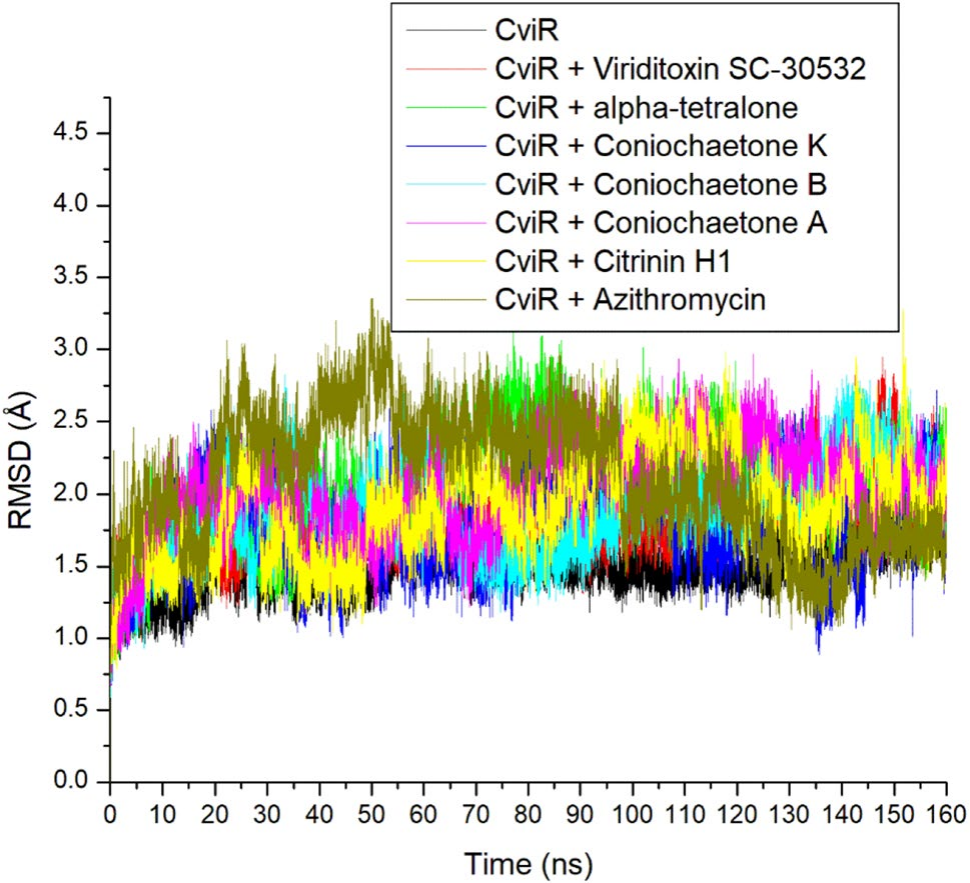
Comparative RMSD plot of alpha-carbon, top six compounds, and Azithromycin against CviR of *C. violaceum* over 160 ns MD simulation period.

The RMSD trajectory depicts the divergence of the protein–ligand complex structure from its apo structure over time. The lower the value, the higher the stability of the protein–ligand complex^26^. In this study, after 10 ns equilibration, each system increased RMSD to 30 ns. An increase in stability to 60 ns was observed for the entire system, except for Azithromycin-CviR, which they maintained with minimal sway through the simulation (Fig. 6). Azithromycin only had a better reduction in RMSD, meaning better stability after 90 ns of simulation. These observations affected the overall average RMSD values, where Azithromycin (2.09 Å) had the highest average RMSD value and thus suggested its lesser stability with CviR relative to the top-ranked investigated compounds (Table 2). Of the top 6 compounds, Coniochaetone K had the lowest RMSD value, suggesting that the compound had a better advantage as an inhibitor of CviR. Compared to the apo-CviR, the binding of CviR had an increasing effect on the RMSD value. However, the RMSD values of all examined compounds (between 1.77 and 2.06 Å) fall within the limit of < 3 Å (Table 2) and therefore indicate a good deviation^27^. Furthermore, the RMSD values obtained from this study fall between 2 and 4 Å reported for the lead quorum sensing inhibitors obtained from *Meliadubia* bark methanolic extract by Ahmad et al.^28^. The RMSD plot showed that both Catechin and Nakinadine B were found stable during 50 ns simulation time with the CviR protein^23^.

**Table 2:** RMSD, RMSF, RoG, SASA, and number of H-bonds of top-ranked compounds against CviR of *C. violaceum* after 160 ns MD simulation.

Ligands	RMSD (Å)	RMSF (Å)	RoG (Å)	SASA (Å)	Intramolecular H-bond
Unbound CviR	1.49 ± 0.15	1.24 ± 0.52	16.07 ± 0.09	9924.08 ± 242.72	91.74 ± 6.62
Coniochaetone A	2.01 ± 0.31	1.23 ± 0.59	16.21 ± 0.12	9865.82 ± 248.22	91.44 ± 6.53
Coniochaetone B	1.90 ± 0.31	1.24 ± 0.66	16.13 ± 0.10	9837.93 ± 245.79	92.70 ± 6.45
Coniochaetone K	1.77 ± 0.30	1.10 ± 0.58	16.21 ± 0.10	10,050.80 ± 238.61	91.82 ± 6.71
Viriditoxin SC-30532	1.79 ± 0.22	1.26 ± 0.58	16.27 ± 0.11	10,236.71 ± 271.44	93.08 ± 6.76
Citrinin H1	1.93 ± 0.34	1.27 ± 0.64	16.23 ± 0.12	10,297.42 ± 264.52	86.98 ± 6.49
(3*S*)-3,8-Dihydroxy-6,7-dimethyl-alpha-tetralone	2.06 ± 0.36	1.44 ± 0.71	16.21 ± 16.21	10,137.81 ± 320.28	89.96 ± 6.65
Azithromycin	2.09 ± 0.43	1.34 ± 0.65	16.47 ± 0.12	10,388.94 ± 278.36	92.57 ± 6.94

### RMSF

**Figure 7:**
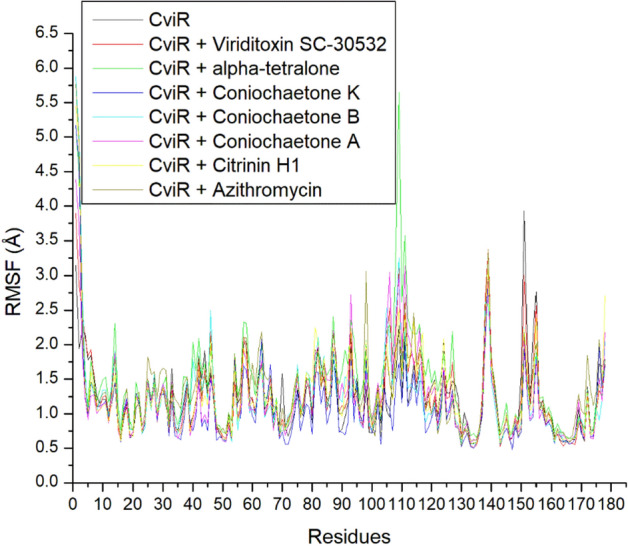
Comparative RMSF plot of alpha-carbon, top six compounds, and Azithromycin against CviR of *C. violaceum* over 160 ns MD simulation period.

The RMSF quantifies the average position of protein residues over time from their reference position^29,30^. As a result, it analyzes the structural parts that vary most or least within a protein, thereby providing information on the stability of formed intra-molecular and inter-molecular bonds. Less variation in residues means stronger bond formation and vice versa for higher residue fluctuation. In this study, the higher residues fluctuations in all the systems between 105 and 115, 135 and 145, and 150 and 155 were observed during the simulation, which suggests lesser capabilities of these residues to form stable bonds intra- and inter-molecularly (Fig. 7). The highest flexibility was observed in (3S)-3,8-Dihydroxy-6,7-dimethyl-alpha-tetralone-CviR complex (1.44 Å), and except for this complex, all other complexes formed by the top-ranked compounds with CviR cause lesser flexibility of CviR residues than Azithromycin (Table 2) which suggest their better thermodynamic compatibility with the target. Of the top-ranked compounds, Coniochaetone K complexed with CviR had the least (1.10 Å) RMSF value (Table 2). This finding was consistent with the current study's RMSD and binding free energy results and supported the benefit of the drug as a CviR inhibitor.

### RoG

**Figure 8:**
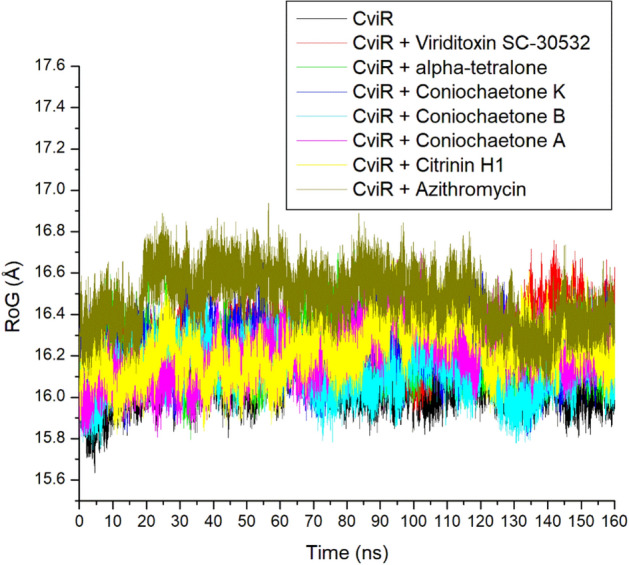
Comparative ROG plot of alpha-carbon, top six compounds, and Azithromycin against CviR of *C. violaceum* over 160 ns MD simulation period.

The RoG indicates the compactness and size of the protein structure, which measures the distance between the center of a protein mass and its two termini^31^. The lesser the RoG value, the more compact and stable the complex. In this study, the apo-CviR, Azithromycin complex, and top-ranked compounds complexes with CviR showed stability around 15.8–16.8 Å (Fig. 8). The average RoG of apo-CviR (16.07 Å) was marginally lower than that of the bounded systems (16.13–16.47 Å), with Azithromycin having the highest value (Table 2). The closely related RoG values observed between the apo-CviR and the bound systems demonstrated the negligible binding effect on protein folding. They showed that the binding of CviR by top-ranked compounds of *Cladosporium* spp. and Azithromycin does not induce entropy in the protein. The relatively lower RoG value of the high-ranking compounds, particularly Coniochaetone B (16.13 Å) against CviR, compared to Azithromycin-CviR (16.47 Å), might suggest some advantages of the top compounds as inhibitors of CviR compared to Azithromycin, despite the higher affinity assessment of Azithromycin. This observation was consistent with this study's RMSD and RMSF results, identifying the top-ranked compounds as promising anti-quorum sensing inhibitors.

### SASA

**Figure 9:**
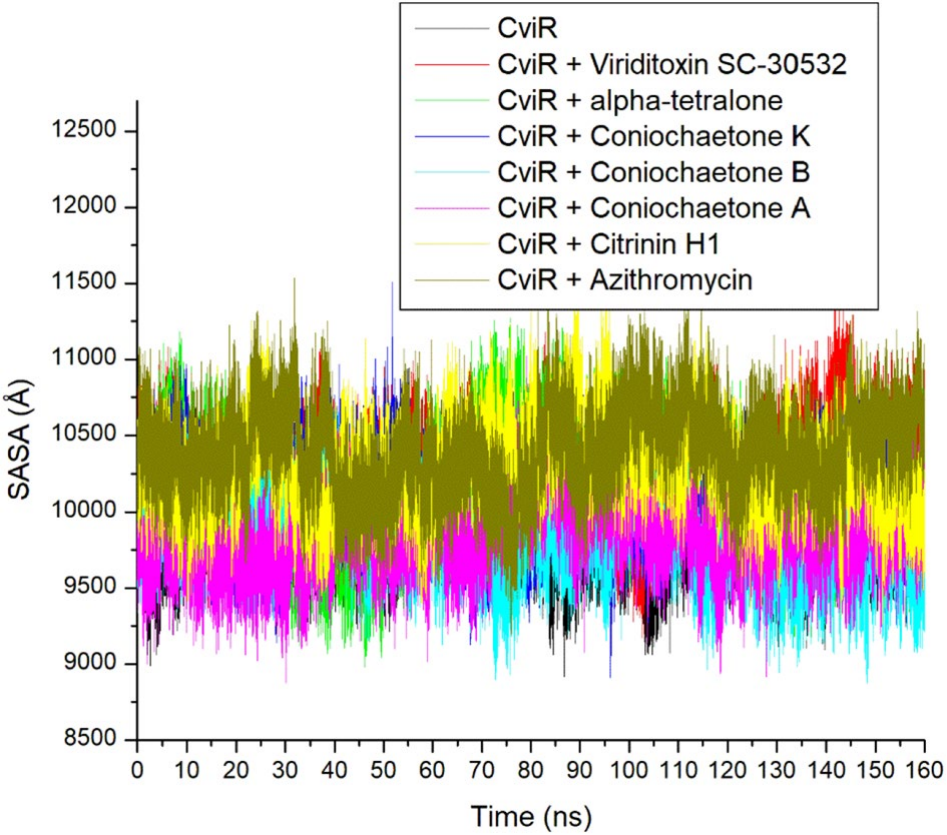
Comparative SASA plot of alpha-carbon, top six compounds, and Azithromycin against CviR of *C. violaceum* over 160 ns MD simulation period.

The changes in solvent accessibility of protein residue, which can be determined by computing the SASA values of protein, are a deciding factor in protein folding and stability studies^32^. It describes the area of protein exposed enough to interact with the adjacent solvent molecules. The lesser the SASA value, the lesser the protein volume and surface area, which could suggest better stability. Like the RoG, the apo-CviR, Azithromycin complex, and top-ranked compounds complexes with CviR showed stability during the simulation between 9000 and 11,000 Å (Fig. 9). The average SASA value for all the systems ranges between 9865.82 and 10,388.94 Å, with Azithromycin having the highest value (Table 2). The closely related SASA values observed between the apo-CviR and the bound systems agree with this study's RoG findings, which could mean the negligible effect of binding on protein folding and demonstrate that CviR ligand binding does not induce entropy in the protein. The lesser SASA value observed with the high-ranked compounds relative to Azithromycin, while consistent with other thermodynamic metrics investigated in the study, further highlights the high-ranking compounds' thermodynamic compatibility, especially Coniochaetone B, as promising anti-quorum sensing inhibitors.

### Number of H-bonds

**Figure 10:**
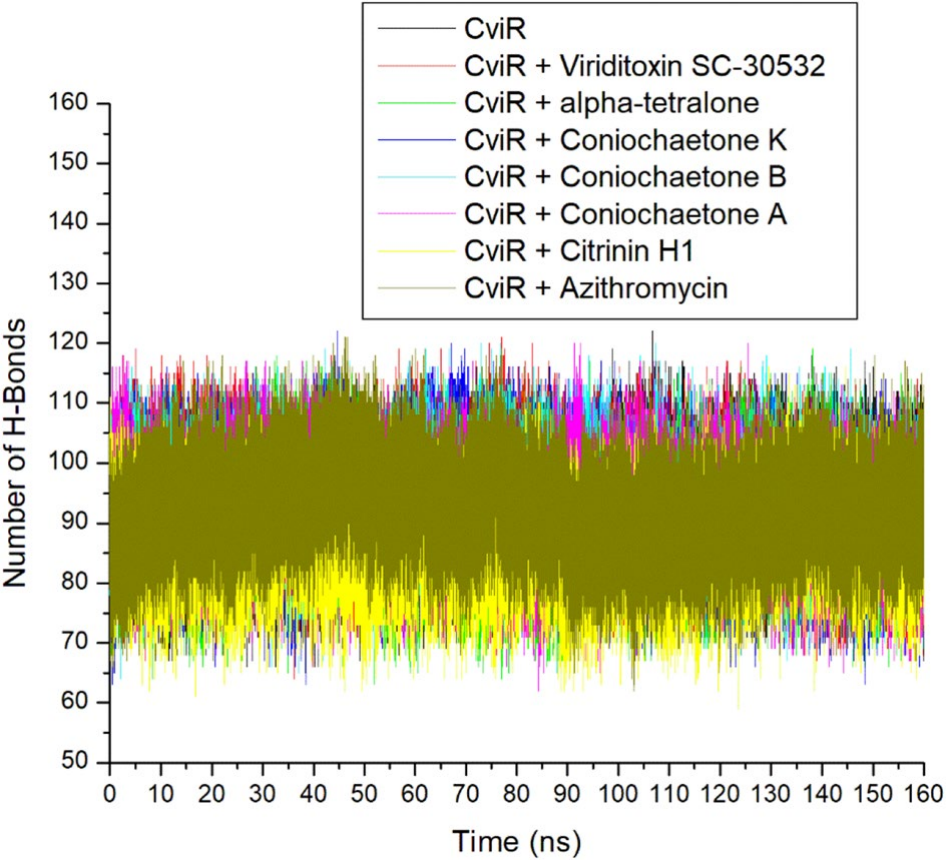
Time evolution of the number of H-bond plots of alpha-carbon, top six compounds, and Azithromycin against CviR of *C. violaceum* over 160 ns MD simulation period.

The hydrogen (H) bonds are the most important interactions which play vital roles in protein structural stability, molecular recognition, the permeability of drugs, and enzyme catalysis^33^. The ability to form hydrogen bonds can enhance the solubility of the drug and establish specific interactions with biomolecular targets, thereby driving the potent selectivity and binding of drugs. A stable swaying of intra-molecular hydrogen bonds (between 65 and 115) in apo-CviR and the bounded CviR complexes during the 160 ns simulation period, suggesting thermodynamic orderliness in all the systems (Fig. 10). This observation agrees with the other MD simulation metrics assessed in this study. The intra-molecular H-bonds have numbers ranging between 86.98 and 93.08, with Viriditoxin SC-30532 having the highest value (Table 2). Generally, while demonstrating the thermodynamic compatibility of Azithromycin and the high-ranked compounds of *Cladosporium* spp. with CviR, this observation had little effect in distinguishing the higher free energy of binding observed with Azithromycin relative to the other compounds studied. Recently, Chaieb et al.^23^ have reported that Catechin and Nakinadine B were found to have a stable interaction during 50 ns MD simulation time, identifying them as effective quorum sensing antagonists against the *C. violaceum*.

### Binding free energy calculations

As a result of virtual screening limitations relating to lack of refinement, the docking scores obtained for the top-scoring compounds were rescored using the MM/GBSA technique^34^. The free energy of binding between the high-ranked ligands and CviR was calculated. A higher negative binding free energy value indicates better affinity, while lower negative values indicate otherwise. In this study, Azithromycin (– 44.20 kcal/mol) had a higher negative binding free energy value than the best-ranked compounds of *Cladosporium* spp., with scores ranging between – 26.49 and – 30.87 kcal/mol (Table 3). Generally, these scores are lesser than the – 58.33 kcal/mol reported for Catechin by Chaileb et al.^23^ against CviR. However, it is worth noting that Chaileb et al.^23^ performed only 50 ns MM/GBSA computation of Catechin-CviR compared to the 160 ns exploited in this study to ensure efficient binding of the protein for proper inactivation. The importance of extended MD simulation that can match the time scale of biological processes was recognized by Hospital et al.^24^ as one of the features of selecting effective biomolecules, thus the more extended simulation period in this study. Azithromycin having the highest negative binding free energy score after a 160 ns simulation period contradicts the observation of the docking study where the top six compounds showed better affinities for CviR. However, this observation pinpoints the advantage of subsequent refinement of virtual screening study in selecting active molecules for further in vitro and in vivo drug development. The higher negative binding free energy score of Azithromycin compared with the investigated top-six compounds signifies the better affinity of Azithromycin for CviR and hence a better inhibitor of the target. Of the top-six compounds, Coniochaetone K had the highest negative binding free energy score at – 30.87 kcal/mol, slightly compared with – 30.23 kcal/mol observed for Coniochaetone A (Table 3). This observation pinpoints Coniochaetone K and Coniochaetone A as the best compounds from *Cladosporium* spp. with the capability to inhibit quorum sensing via the inactivation of CviR.

**Table 3:** Energy components (kcal/mol) of top-ranked compounds against CviR of *C. violaceum* after 160 ns MD simulation.

Ligands	ΔE_vdW_	ΔE_elec_	ΔG_gas_	ΔG_solv_	ΔG_bind_
Coniochaetone A	– 32.85 ± 2.56	– 12.75 ± 3.89	– 45.61 ± 5.06	15.37 ± 2.83	– 30.23 ± 3.03
Coniochaetone B	– 34.25 ± 1.97	– 8.80 ± 5.74	– 43.05 ± 6.00	14.83 ± 3.59	– 28.21 ± 3.05
Coniochaetone K	– 32.09 ± 2.93	– 25.86 ± 3.58	– 57.95 ± 3.71	27.07 ± 2.18	– 30.87 ± 2.82
Viriditoxin SC-30532	– 42.03 ± 6.58	– 9.13 ± 7.75	– 51.17 ± 11.30	23.15 ± 7.44	– 28.01 ± 6.16
Citrinin H1	– 37.50 ± 3.87	– 5.63 ± 5.57	– 43.14 ± 7.70	16.65 ± 4.63	– 26.49 ± 4.64
(3*S*)-3,8-Dihydroxy-6,7-dimethyl-alpha-tetralone	– 30.16 ± 2.29	– 9.43 ± 5.35	– 39.59 ± 5.26	13.03 ± 2.94	– 26.56 ± 2.99
Azithromycin	– 43.59 ± 5.98	– 111.65 ± 31.48	– 167.15 ± 34.45	122.95 ± 30.91	– 44.20 ± 6.98

*ΔE*_*vdW*_ van der Waals energy, *ΔE*_*elec*_ Electrostatic energy, *ΔE*_*gas*_ Gas phase free energy, *ΔG*_*sol*_ Solvation free energy, *ΔG*_*bind*_ Total binding free energy.

### Bonds analysis of interaction plots of top-ranked compounds against CviR over 160 ns MD simulation period

**Figure 11:**
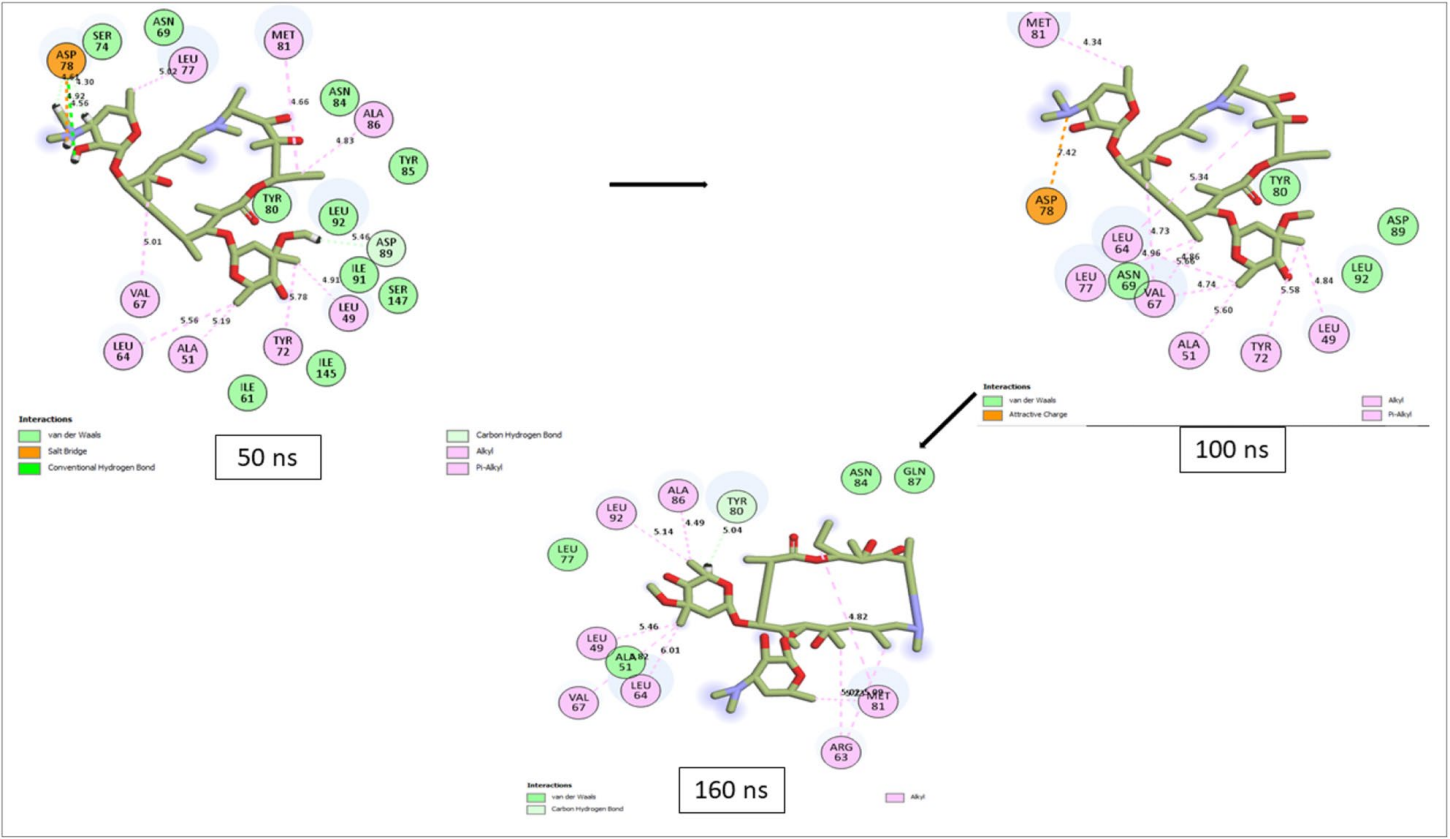
Interactions plot of Azithromycin at different timeframes against CviR of *C. violaceum* during 160 ns MD simulation.

**Figure 12:**
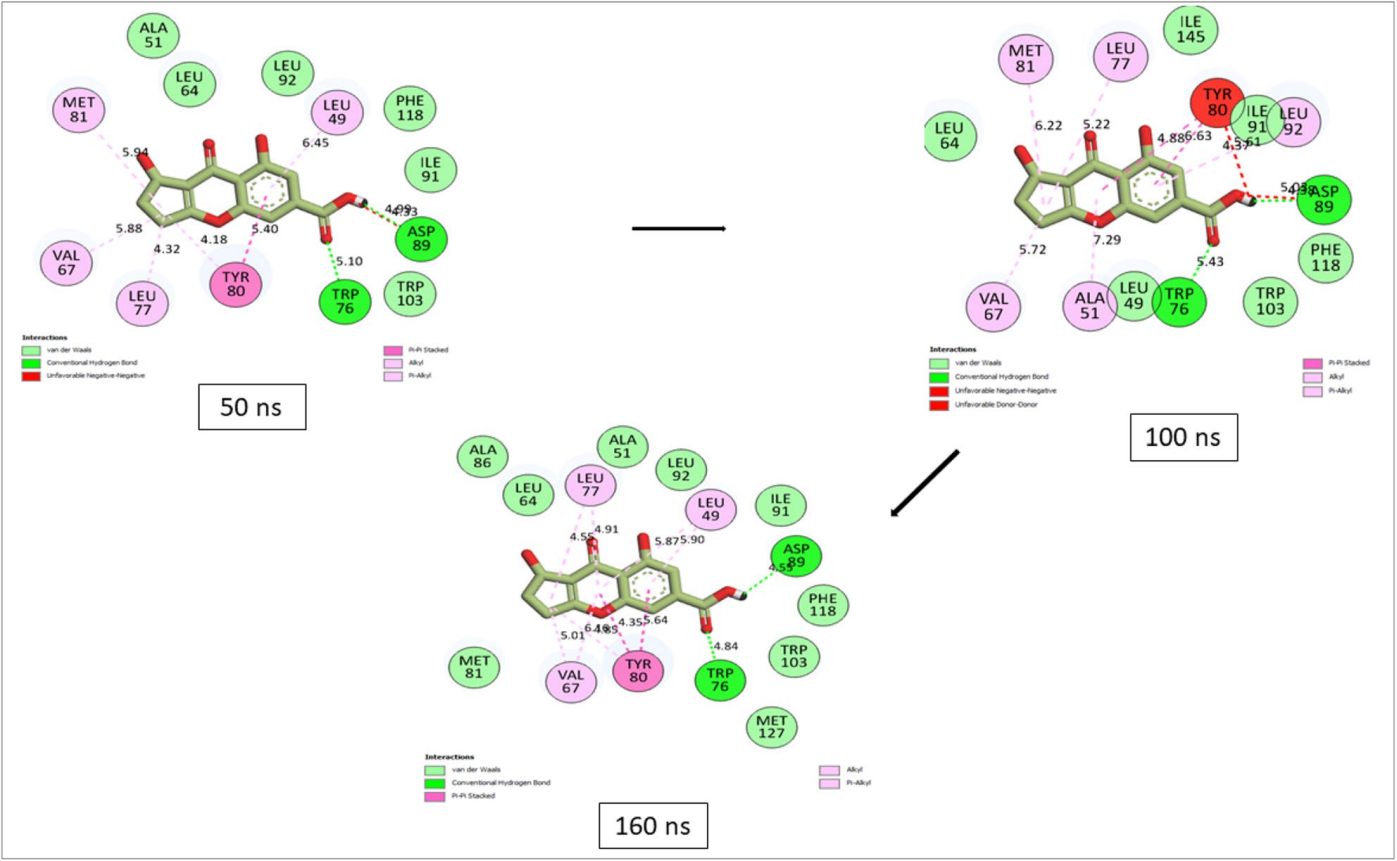
Interactions plot of Coniochaetone K at different timeframes against CviR of *C. violaceum* during 160 ns MD simulation.

Numerous thermodynamic factors, such as flexibility, stability, compactness, and the interactions of proteins with their key amino acids, affect the capacity of ligands to bind to and inactivate the target protein^30,35,36^. Consequently, the study analyzed the length, number, and type of binding interactions formed by the best compounds with CviR at various periods (Supplementary Figs. S1–S7). The plot of interactions at the different timeframes for Azithromycin and compound (Coniochaetone K) with the lowest binding free energy against CviR of *C. violaceum* was presented in Figs. 11 and 12, respectively. Among the top-six compound from *Cladosporium* spp. investigated in this study, Coniochaeton K, with the lowest binding free energy against CviR, had the second lowest number of total interactions at 50 ns; however, as the simulation advanced, the number of interactions between Coniochaetone K and CviR increased, and at 160 ns, Coniochaetone K had 20 interactions with CviR, the second lowest after Coniochaetone B (21 interactions) (Table 4; Fig. 11). This observation points to the improvement in the interactions of Coniochaeton K with CviR of *C. violaceum* as the period of the simulation increases and could have contributed to the high affinity observed with the compounds relative to other investigated compounds of *Cladosporium* spp. with CviR.

**Table 4:** Bond analysis at different timeframes against CviR active site during the 160 ns simulation period.

Complex	Total number of interactions (distance Å)			Total number of H-bonds (distance Å)			Total No. of other important interactions (distance Å)			Number of conserved H-bond residues at each of the time frame
	50 ns	100 ns	160 ns	50 ns	100 ns	160 ns	50 ns	100 ns	160 ns	
Coniochaetone A	14 (5.27)	16 (5.25)	19 (5.46)	1 (4.10)	1 (4.14)	3 (4.62)	6 (5.44)	8 (5.39)	9 (5.74)	1 (Ser147)
Coniochaetone B	19 (5.47)	17 (5.37)	21 (5.61)	1 (4.40)	2 (4.45)	3 (4.79)	9 (5.59)	8 (5.60)	10 (5.86)	1 (Thr132)
Coniochaetone K	15 (5.17)	17 (5.52)	20 (5.14)	2 (4.71)	2 (4.88)	2 (4.69)	7 (5.30)	9 (5.66)	9 (5.24)	2 (Asp89, Trp76)
Viriditoxin SC-30532	20 (5.11)	19 (5.06)	18 (5.15)	2 (3.86)	1 (3.83)	3 (4.74)	9 (5.39)	11 (5.18)	9 (5.29)	–
Citrinin H1	18 (5.11)	18 (4.76)	19 (5.12)	2 (3.74)	3 (3.77)	3 (4.74)	10 (5.39)	9 (5.09)	10 (5.24)	1 (Asn84)
(3S)-3,8-Dihydroxy-6,7-dimethyl-alpha-tetralone	20 (5.07)	22 (5.33)	15 (5.31)	–	2 (4.86)	1 (5.07)	11 (5.07)	16 (5.39)	7 (5.35)	–
Azithromycin	23 (4.98)	15 (5.27)	14 (4.95)	4 (4.73)	–	1 (5.04)	9 (5.09)	13 (5.27)	9 (4.94)	–

Furthermore, Coniochaeton K in complex with CviR had two H-bond interactions with Asp89 and Trp76 that were consistent throughout the simulation, an observation that was absent with other compounds investigated in this study. This observation was consistent with the RMSF results of this study, where the Coniochaeton K-CviR complex had the least fluctuation suggesting stable bond formation. Coniochaetone A, Coniochaetone B, and Citrinin H1 only had one consistent H-bond with Ser147, Thr132, and Asn84, respectively, while other investigated compounds had no stable H-bond residues during the simulations (Table 4). Due to their exceptionally strong inter-molecular interactions, H-bond interactions have become a very strong non-covalent bond interaction in drug design, and its consistency in simulations may, therefore, greatly aid stability^35^. Generally, Azithromycin showed higher binding free energy with CviR than the top-ranked metabolite of *Cladosporium* spp. Following bond analysis, Azithromycin had 23 and 4 H-bond interactions after 50 ns. However, this interaction reduced gradually as the simulation progressed. After 160 ns, the Azithromycin-CviR complex had 14 total interactions and 1 H-bond contact with CviR, the lowest among the investigated compounds in this study (Table 4; Fig. 12).

The high number of H-bonding interactions before 50 ns could have contributed to the observed higher free energy of binding of Azithromycin against CviR. However, the reduction in interactions after 50 ns, while corroborating the RMSF findings of this study on the slightly high fluctuation of CviR residues (indicating less stable bonds) when complexed with Azithromycin, points to the lesser affinity of Azithromycin for CviR after 50 ns. Among the top-ranked compounds of *Cladosporium* spp. only (3S)-3,8-Dihydroxy-6,7-dimethyl-alpha-tetralone and Viriditoxin SC-30532 had a decrease in interactions after 50 ns. This observation had an impact on their binding free energy as (3S)-3,8-Dihydroxy-6,7-dimethyl-alpha-tetralone and Viriditoxin SC-30532 had lower affinity compared to Coniochaetone A, B, and K. Finally, the binding analysis points to the promising interactions of Coniochaetone A, B, and K with CviR with at least one consistent H-bond contact during the 160 ns simulation, and therefore points to their advantage as a promising inhibitor of CviR. However, Azithromycin had the lowest number of interactions at 160 ns. The binding analysis of the complex indicates reduced interactions after 50 ns, which may compromise its potential to inhibit the protein over a long period.

### Pharmacokinetics and toxicity evaluation

The physicochemical and pharmacokinetics properties of selected compounds were predicted after molecular docking and MD simulation analyses to aid in the search for novel drugs with intriguing polypharmacological profiles. The present study computed the physicochemical parameters, such as molecular weight, partition coefficient (log P), hydrogen bond acceptors, hydrogen bond donors, rotatable bonds, and polar surface area (PSA), to determine which drugs had a high oral bioavailability^37^. Molecules that meet the following criteria: molecular weight ≤ 500 mg/mol, log P ≤ 5, hydrogen bond acceptors ≤ 10, hydrogen bond donors ≤ 5, rotatable bond count ≤ 10, and PSA ≤ 140, suggest such molecule could penetrate cell membrane with improved oral bioavailability. The physicochemical properties of potential compounds from *Cladosporium* spp. and positive control are represented in Table 5. Compared to Azithromycin, the potential compounds became apparent to satisfy most drug-likeness criteria, rendering them potential drug candidates.

**Table 5:** Physicochemical properties of potential compounds from *Cladosporium* spp. and positive control.

Compound name	Coniochaetone			Viriditoxin SC-30532	Citrinin H1	(3S)-3,8-Dihydroxy-6,7-dimethyl-alpha-tetral-one	Azithromycin
	A	B	K				
Molecular weight (D)	230.219	232.235	262.217	630.602	442.508	206.241	749
log P	1.93592	1.78662	1.1764	4.6208	3.69712	1.49884	1.9007
Rotatable bonds	0	0	1	7	3	0	7
Hydrogen bond acceptors	4	4	5	12	7	3	14
Hydrogen bond donors	1	2	3	4	1	2	5
Polar surface area (Å^2^)	96.489	97.122	106.078	261.227	187.278	88.365	311.558

Molecular lipophilicity is the ability of a compound to mix more readily with an oil phase rather than with water and is usually calculated through atom-based log P calculations^38^. All six potential compounds and Azithromycin were predicted to have strong lipophilicity as they have log P ≤ 5, suggesting strong absorption and membrane permeation. The PSA is one of the primary factors of fractional intestinal absorption that indicates the compounds' intestinal permeability and absorption properties^39^. The PSA of Viriditoxin SC-30532, Citrinin H1, and Azithromycin were > 140 Å^2^ proposing their strong polarity and the relativities difficulties for absorption in the body. The PSA of Coniochaetone A, Coniochaetone B, Coniochaetone K, and (3S)-3,8-Dihydroxy-6,7-dimethyl-alpha-tetralonewere all < 140 Å^2^ indicated that these compounds would demonstrate good oral absorption and membrane permeability.

Additionally, the pharmacokinetic properties of prospective substances are significantly influenced by the prediction of their physicochemical descriptors^18^. The predicted pharmacokinetic properties of substances such as ADMET are essential for developing innovative drugs with desirable biological activity. The pkCSM results predicted the ADMET properties of potential compounds from *Cladosporium* spp. and positive control (Table 6). The physiological parameters to predict drug absorption include membrane permeability [as measured by the permeability coefficient of the human colon cancer cell line (Caco-2)], intestinal absorption, skin permeability, P-glycoprotein inhibitor, and P-glycoprotein substrate^40^. The human intestinal permeability and absorption of drugs are often estimated using permeability coefficients across Caco-2 monolayers^41^. Generally, when the predicted permeability value is > 0.9, it suggests that the compound has a Papp coefficient of > 8 × 10^−6^ cm/s; hence, such a compound is considered to show a high Caco-2 permeability. Results from this study showed that Coniochaetone A, Coniochaetone B, and (3S)-3,8-Dihydroxy-6,7-dimethyl-alpha-tetralone possess a predicted permeability value of > 0.9 which means that they have a high Caco-2 permeability compared to Azithromycin with < 0.9.

**Table 6:** Pharmacokinetics properties of potential compounds from *Cladosporium* spp. and positive control.

Properties	Compound Name	Coniochaetone			Viriditoxin SC-30532	Citrinin HI	(35)-3,8-Dihydroxy-6,7-dimethyl-alpha-tetralone	Azithromycin
		A	B	K				
Absorption	Water solubility (log mol/L)	− 2.688	− 2.878	− 2.772	− 3.104	− 4.348	− 2.33	− 4.133
	Caco-2 permeability (log Papp in 10^−6^ cm/s)	0.975	0.959	-0.17	0.263	0.855	1.164	− 0.211
	Human intestinal absorption (% absorbed)	95.42	94.574	60.689	93.204	83.409	94.522	45.808
	Skin permeability (log Kp in cm/h)	− 2.857	− 2.796	-2.735	− 2.735	− 2.896	− 3.412	− 2.742
	P-glycoprotein substrate	No	Yes	Yes	Yes	No	No	Yes
	P-glycoprotein I inhibitor	No	No	No	Yes	Yes	No	Yes
	P-glycoprotein II inhibitor	No	No	No	Yes	No	No	No
Distribution	Human VDss (log L/kg)	0.059	0.25	− 1.588	− 1.257	0.462	0.236	− 0.214
	Human fraction unbound (Fu)	0.344	0.379	0.375	0.333	0.106	0.478	0.512
	B1313 permeability (log BB)	− 0.004	0.126	− 1.025	− 2.157	− 0.677	− 0.285	− 1.857
	CNS permeability (log PS)	− 2.04	− 2.116	− 3.046	− 4.009	− 2.856	− 2.843	− 3.777
Metabolism	CYP2D6 substrate	No	No	No	No	No	No	No
	CYP3A4 substrate	No	No	No	No	Yes	No	Yes
	CYP IA2 inhibitor	Yes	Yes	No	No	No	Yes	No
	CYP2C19 inhibitor	No	No	No	No	No	No	No
	CYP2C9 inhibitor	No	No	No	No	No	No	No
	CYP2D6 inhibitor	No	No	No	No	No	No	No
	CYP3A4 inhibitor	No	No	No	No	Yes	No	No
Excretion	Total clearance (log mL/min/kg)	0.444	0.401	0.563	− 0.606	0.516	0.16	-0.424
	Renal OCT2 substrate	No	No	No	No	No	No	No
Toxicity	AMES toxicity	Yes	No	No	No	No	No	No
	Human Max. tolerated dose (log mg/kg/day)	0.081	0.274	1.109	0.378	0.548	0.537	1.027
	hERG I inhibitor	No	No	No	No	No	No	No
	hERG II inhibitor	No	No	No	Yes	No	No	No
	Oral rat acute toxicity (LD50) (mol/kg)	2.096	2.134	2.396	2.64	2.569	2.002	2.769
	Oral rat chronic toxicity (LOAEL) (log mg/kg bw/day)	1.708	1.48	1.945	1.386	2.151	1.892	1.991
	Hepatotoxicity	No	No	No	No	No	No	Yes
	Skin sensitization	No	No	No	No	No	No	No
	*Tetrahymena pyriformis *toxicity (log µg/L)	0.922	0.624	0.285	0.285	0.293	0.558	0.285
	Minnow toxicity (log mM)	0.908	1.728	1.714	− 2.156	0.237	2.466	7.8

In the process of developing new pharmaceutical compounds, human intestine absorption has a substantial impact on the bioavailability of the drugs^42^. In response to human intestinal absorption, an absorbance value < 30% is considered poorly absorbed. Findings from this study showed that all top-ranked compounds had a higher absorption rate than Azithromycin. The human skin permeability is the rate at which the chemicals pass through the stratum corneum, the epidermis' outer layer^43^. The skin permeability coefficient (log Kp) value is frequently employed to assess the transport of small substances through the stratum corneum. A compound is said to have low skin permeability if its log Kp value is > – 2.5 cm/h. All top-ranked compounds and Azithromycin had high skin permeability as they had a < – 2.5 cm/h log Kp value. One of the drug transporters in charge of regulating the absorption and efflux of diverse medicines is the P-glycoprotein^44^. P-glycoprotein transports the P-glycoprotein substrates out of the cell, whereas P-glycoprotein inhibitors prevent P-glycoprotein transport. In this study, Coniochaetone B, Coniochaetone K, Viriditoxin SC-30532, and Azithromycin were predicted to be P-glycoprotein substrates suggesting they could be actively excreted from the cells. In addition, Coniochaetone A, Coniochaetone B, Coniochaetone K, and (3S)-3,8-Dihydroxy-6,7-dimethyl-alpha-tetralone were predicted non-inhibitor of P-glycoprotein compared to Azithromycin that could inhibit the protein.

The second stage of pharmacokinetics is the distribution of the drug, which is a crucial process of distributing the drug to reach its intended target site of action in the human body via the blood flow^45^. The criteria used to comprehend drug distribution in human tissues in vivo consist of the volume of distribution (VDss), human fraction unbound, blood–brain barrier (BBB), membrane permeability (logBB), and central nervous system (CNS) permeability^46^. The distribution volume where the drug may theoretically distribute in the body along with the blood flow is the apparent VDss^47^. The distribution volume is relatively low if the VDss value is < 0.71 L/kg (log VDss < – 0.15). However, the VDss is considered relatively high if the VDss value is > 2.81 L/kg (log VDss > 0.45). The predicted results showed that the VDss of Coniochaetone K, Viriditoxin SC-30532, and Azithromycin were low as they had a VDss value < – 0.15, but Citrinin H1 was considered high. The drug's ability to enter the human brain is an essential factor that must be regarded as a compound whose pharmacological action is in the human brain to enhance efficacy and reduce drug toxicity^48^. The compounds are considered to cross the BBB easily if the logBB value is > 0.3; if the logBB value is < – 1, it suggests the compound won't cross the BBB easily. All the top-six compounds and Azithromycin were predicted to cross the BBB. In addition, Coniochaetone K, Viriditoxin SC-30532, and Azithromycin, with log PS value ≤ – 3, suggest they cannot penetrate the CNS, while Coniochaetone A, Coniochaetone B, Citrinin H1, and (3S)-3,8-Dihydroxy-6,7-dimethyl-alpha-tetralone may penetrate the CNS.

The metabolism of drugs is predicted based on the computational model of cytochrome P450 (CYP) for substrate or inhibitor (CYP1A2, CYP2C19, CYP2C9, CYP2D6, and CYP3A4). The CYP system plays the most important role in drug metabolism in the liver by converting the drugs from a hydrophobic state to an excretable hydrophilic state^49^. Findings from this study showed that Coniochaetone A, Coniochaetone B, Coniochaetone K, Viriditoxin SC-30532, and (3S)-3,8-Dihydroxy-6,7-dimethyl-alpha-tetralone were not substrates for the two main subtypes of CYP (CYP2D6 and CYP3A4). However, Citrinin H1 and Azithromycin were substrates for CYP3A4, meaning they could be metabolized CYP3A4—Coniochaetone A, Coniochaetone B, and (3S)-3,8-Dihydroxy-6,7-dimethyl-alpha-tetralone were predicted only to inhibit CYP1A2. Citrinin H1 and Azithromycin were found to be CYP3A4 inhibitors. This indicated that one or more CYP isoenzymes could metabolize all top-six compounds and Azithromycin in the liver.

Drug excretion involves removing pharmaceuticals from the body in their original form or as active or inactive biotransformed metabolites^50^. The renal organic cation transporter 2 (OCT2) substrate and total clearance model made up the parameters of drug excretion acquired from the pkCSM^51^. The predicted results showed that Coniochaetone A, Coniochaetone B, Coniochaetone K, and Citrinin H1 had a higher total clearance value than Azithromycin, suggesting that Coniochaetone A, Coniochaetone B, Coniochaetone K, and Citrinin H1 could be excreted more easily from the body than Azithromycin. The OCT2 is a crucial player in drug disposition and renal clearance, which acts as a main renal drug uptake transporter^52^. The pkCSM results showed that all the top-six compounds and Azithromycin were not OCT2 substrates, thus, indicating that they could not be an uptake in the renal and hence will not stay longer in the body. The AMES toxicity, human ether-a-go-go-go-related gene (hERG) inhibitors, acute and chronic toxicity, hepatotoxicity, and skin sensitization are the essential parameters for drug toxicity^53–55^. The Ames test is a powerful bacterial bioassay used to discover potential carcinogens by analyzing their adverse mutagenesis effects on bacteria^56^. The results also indicated that Coniochaetone A could be toxic in the AMES test. But, all six potential compounds and Azithromycin could not inhibit the hERG channel, except Viriditoxin SC-30532, which acts as an hERG II inhibitor. All top-ranked compounds were not hepatotoxic compared to Azithromycin. However, all the top-six compounds and Azithromycin may demonstrate skin sensitization.

## Conclusion

The current study intends to identify effective inhibitors of quorum sensing from microbial sources as an alternative to plant-based antibiotics against *C. violaceum*. The docking analysis showed that CviR protein in *C. violaceum* was inhibited by some potential compounds of *Cladosporium* spp. based on their lower binding energy. The stability and flexibility in CviR complexed with top-ranked compounds were improved during the 160 ns simulation. The pharmacokinetic properties of top-ranked compounds suggested their good oral absorption, membrane permeability, high Caco-2 permeability, and non-hepatotoxicity compared to Azithromycin. Coniochaetone A, Coniochaetone B, Viriditoxin SC-30532, Citrinin H1, and (3S)-3,8-Dihydroxy-6,7-dimethyl-alpha-tetralone and Coniochaetone K had great anti-quorum sensing potential by quorum quenching via interaction of hydrogen bonding and hydrophobic interactions with amino acid residues of CviR protein. Before clinical studies, further in vitro and in vivo studies are required to establish their potential use as future quorum sensing inhibitors to combat *C. violaceum* pathogenesis.

### Supplementary Information

Supplementary Information.

## Data Availability

The data presented in this study are available in this manuscript.
