# Supplementary material for: Exploration of CviR-mediated quorum sensing inhibitors from Cladosporium spp. against Chromobacterium violaceum through computational studies
Source: Sci Rep. 2023 Sep 19;13:15505. doi: 10.1038/s41598-023-42833-4 (PMC10509224; doi:10.1038/s41598-023-42833-4)
Supplement: Supplementary file 1 — Supplementary Information. [file 41598_2023_42833_MOESM1_ESM.docx]

**Supplementary Data**

**Supplementary Table 1.** Molecular docking of bioactive compounds from *Cladosporium* spp. and positive control against CviR protein of *C. violaceum*.

| **Compound Name** | **Binding energy (kcal/mol)** |
| --- | --- |
| Aspidospermidin-20-ol, 1-acetyl-17-methoxy | –6.5 |
| Cladosporine A | –7.4 |
| Cytochalasin D | –7.7 |
| 2-Methylacetate-3,5,6-trimethylpyrazine | –6.9 |
| Lunatoic acid A | –6.6 |
| (6b*S*,7*R*,8*S*)-4,9-Dihydroxy-7,8-dimethoxy-1,6b,7,8-tetra-hydro-2*H*-benzo[J]fluoranthen-3-one | –7.1 |
| (6b*R*,7*R*,8*S*)-7-Methoxy-4,8,9-trihydroxy-1,6b,7,8-tetrahydro-2*H*-benzo[J]fluoranthen-3-one | –7.3 |
| (6b*S*,7*R*,8*S*)-7-Methoxy-4,8,9-trihydroxy-1,6b,7,8-tetrahydro-2*H*-benzo[J]fluoranthen-3-one | –7.0 |
| Coniochaetone A | –9.3 |
| Coniochaetone B | –9.5 |
| Coniochaetone K | –8.5 |
| Cladosporinone | –7.5 |
| Viriditoxin | –7.3 |
| Viriditoxin SC-28763 | –7.9 |
| Viriditoxin SC-30532 | –8.8 |
| Cladospolide F | –6.7 |
| Cladospolide G | –6.8 |
| Ent-Cladospolide F | –6.7 |
| 11-Hydroxy-gamma-dodecalactone | –6.9 |
| Iso-Cladospolide B | –6.7 |
| Citrinin H1 | –8.5 |
| Cladosporin A | –6.6 |
| Cladosporin B | –6.9 |
| Cladosporin C | –7.2 |
| Cladosporin D | –6.2 |
| Cladosporin | –7.2 |
| 5’-Hydroxyasperentin | –7.8 |
| Isocladosporin | –7.1 |
| Cladoscyclitol B | –6.3 |
| 3-Hydroxy-2,4,5-trimethylphenyl 4-[(2,4-dihydroxy-3,6-dimethylbenzoyl)oxy]-2-hydroxy-3,6-dimethylbenzoate | –6.9 |
| 3-Hydroxy-2,5-dimethylphenyl 2,4-dihydroxy-3,6-dimethylbenzoate | –7.3 |
| 3-Hydroxy-2,5-dimethylphenyl 4-[(2,4-dihydroxy-3,6-dimethylbenzoyl)oxy]-2-hydroxy-3,6-dimethylbenzoate | –8.0 |
| (2S)-7,4’-Dihydroxy-5-methoxy-8-(gamma,gamma-dimethylallyl)-flavanone | –7.3 |
| Cladosporamide A | –7.3 |
| Cladocladosin A | –6.8 |
| Cladospolide B | –7.3 |
| 5*R*-Hydroxyrecifeiolide | –6.0 |
| 5*S*-Hydroxyrecifeiolide | –6.1 |
| 5*Z*-7-Oxozeaenol | –6.9 |
| Pandangolide 1 | –5.5 |
| Pandangolide 3 | –6.2 |
| Sporiolide A | –7.4 |
| Sporiolide B | –5.2 |
| Thiocladospolide A | –6.5 |
| Thiocladospolide B | –6.6 |
| Thiocladospolide C | –5.8 |
| Thiocladospolide D | –6.1 |
| Thiocladospolide F | –7.2 |
| Thiocladospolide F *bis* | –7.5 |
| Thiocladospolide G | –5.9 |
| Thiocladospolide G *bis* | –5.8 |
| Thiocladospolide H | –6.5 |
| Thiocladospolide I | –7.7 |
| Thiocladospolide J | –7.3 |
| Zeaenol | –7.1 |
| Cladonaphchrom A | –8.0 |
| Cladonaphchrom B | –7.4 |
| Cladosporol A | –7.5 |
| Cladosporol B | –7.7 |
| Cladosporol C | –7.8 |
| Cladosporol D | –7.2 |
| Cladosporol E | –7.8 |
| Cladosporol F | –7.6 |
| Cladosporol G | –7.4 |
| Cladosporol G *bis* | –8.1 |
| Cladosporol H | –7.9 |
| Cladosporol I | –7.7 |
| Cladosporol J | –7.3 |
| Cladosporone A | –7.7 |
| (3*S*)-3,8-Dihydroxy-6,7-dimethyl-alpha-tetralone | –8.6 |
| Scytalone | –6.6 |
| Anhydrofusarubin | –8.3 |
| Fusarubin methyl ether | –7.6 |
| Altertoxin VIII | –8.1 |
| Altertoxin IX | –7.8 |
| Altertoxin X | –7.8 |
| Altertoxin XI | –7.7 |
| Altertoxin XII | –7.6 |
| Calphostin A | –5.6 |
| Calphostin B | –5.9 |
| Calphostin C | –6.7 |
| Calphostin D | –6.7 |
| Calphostin I | –5.9 |
| Phleichrome | –5.8 |
| Cladospolide E | –6.7 |
| Seco-patulolide A | –6.8 |
| Seco-patulolide C | –6.7 |
| (3*S*,5*S*,11*S*)-Trihydroxydodecanoic acid | –6.7 |
| Cladosporide A | –6.6 |
| Cladosporide B | –7.1 |
| Cladosporide C | –6.7 |
| 3alpha-Hydroxy-pregn-7-ene-6,20-dione | –7.8 |
| Cladodionen | –7.1 |
| Cladosin B | –6.3 |
| Cladosin C | –7.0 |
| Cladosin F | –8.1 |
| Cladosin I | –6.7 |
| Cladosin J | –7.2 |
| Cladosin K | –6.9 |
| Cladosin L | –6.5 |
| Cladosin L *bis* | –7.6 |
| Cladosporicin A | –8.2 |
| *Cladosporium*in I *bis* | –7.5 |
| *Cladosporium*in J *bis* | –7.4 |
| Malettinin A | –7.4 |
| Malettinin B | –8.3 |
| Malettinin C | –7.8 |
| Malettinin E | –6.7 |
| Conioxanthone A | –7.3 |
| 3,8-Dihydroxy-6-methyl-9-oxo-9*H*-xanthene-1-carboxylate | –8.2 |
| Alpha-Diversonolic ester | –7.2 |
| Beta-Diversonolic ester | –8.0 |
| 8-Hydroxy-6-methylxanthone-1-carboxylic acid | –7.1 |
| Methyl 8-hydroxy-6-(hydroxymethyl)-9-oxo-9*H*-xanthene-1-carboxylate | –7.2 |
| Methyl 8-hydroxy-6-methyl-9-oxo-9*H*-xanthene-1-carboxylate | –8.1 |
| 8-(Methoxycarbonyl)-1-hydroxy-9-oxo-9*H*-xanthene-3-carboxylic acid | –7.3 |
| Vertixanthone | –7.9 |
| Acetyl Sumiki's acid | –6.6 |
| 1,1’-Dioxine-2,2’-dipropionic acid | –6.6 |
| 4-*O*-alpha-*d*-Ribofuranose-2-pentyl-3-phemethylol | –6.5 |
| Sumiki's acid | –5.9 |
| Taxol | –7.3 |
| Vermistatin | –6.7 |
| Azithromycin [Positive control] | –7.4 |

**
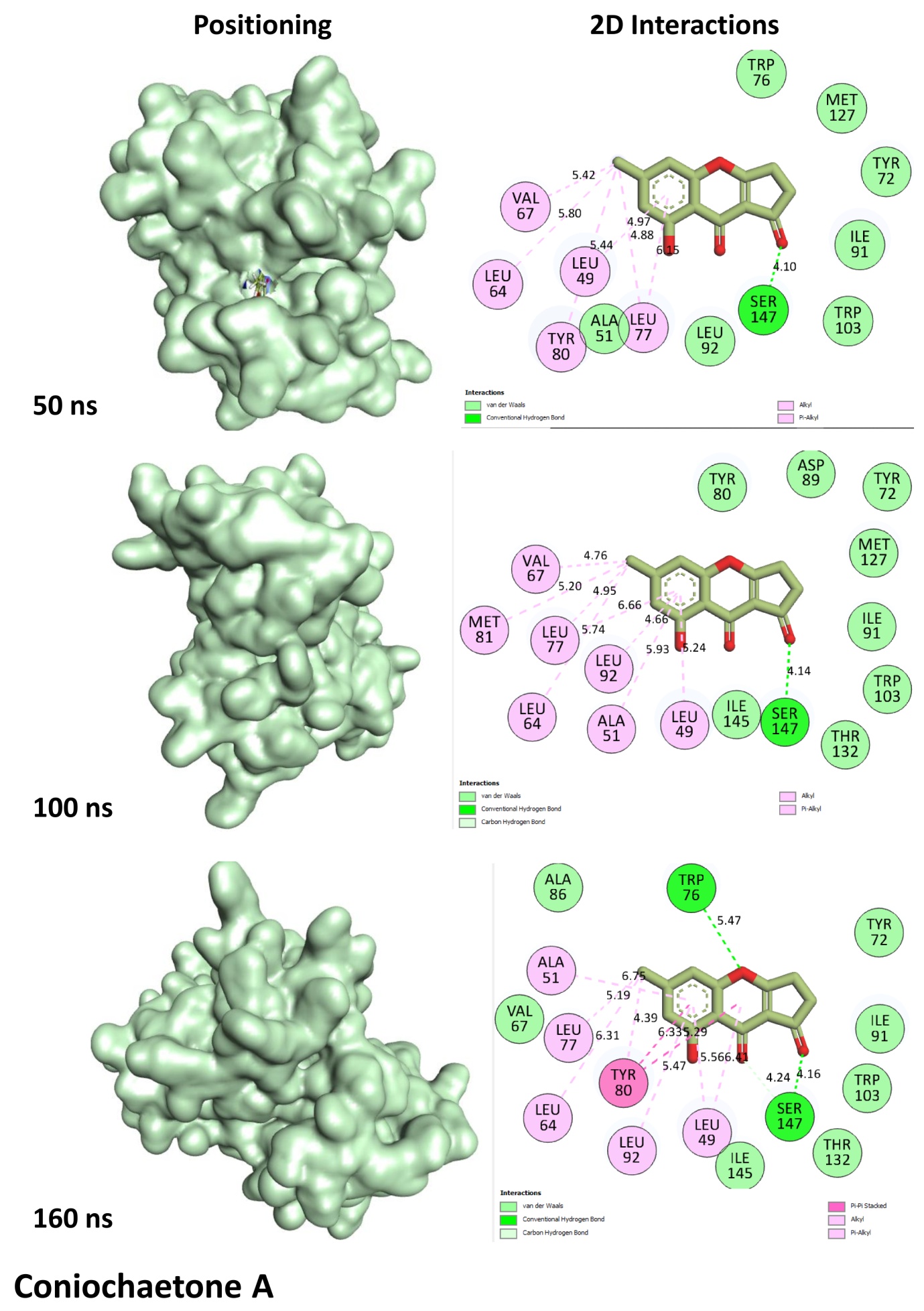
**

**Figure S1.** Interaction plots of Coniochaetone A at different timeframe with CviR during the 160 ns simulation.

**
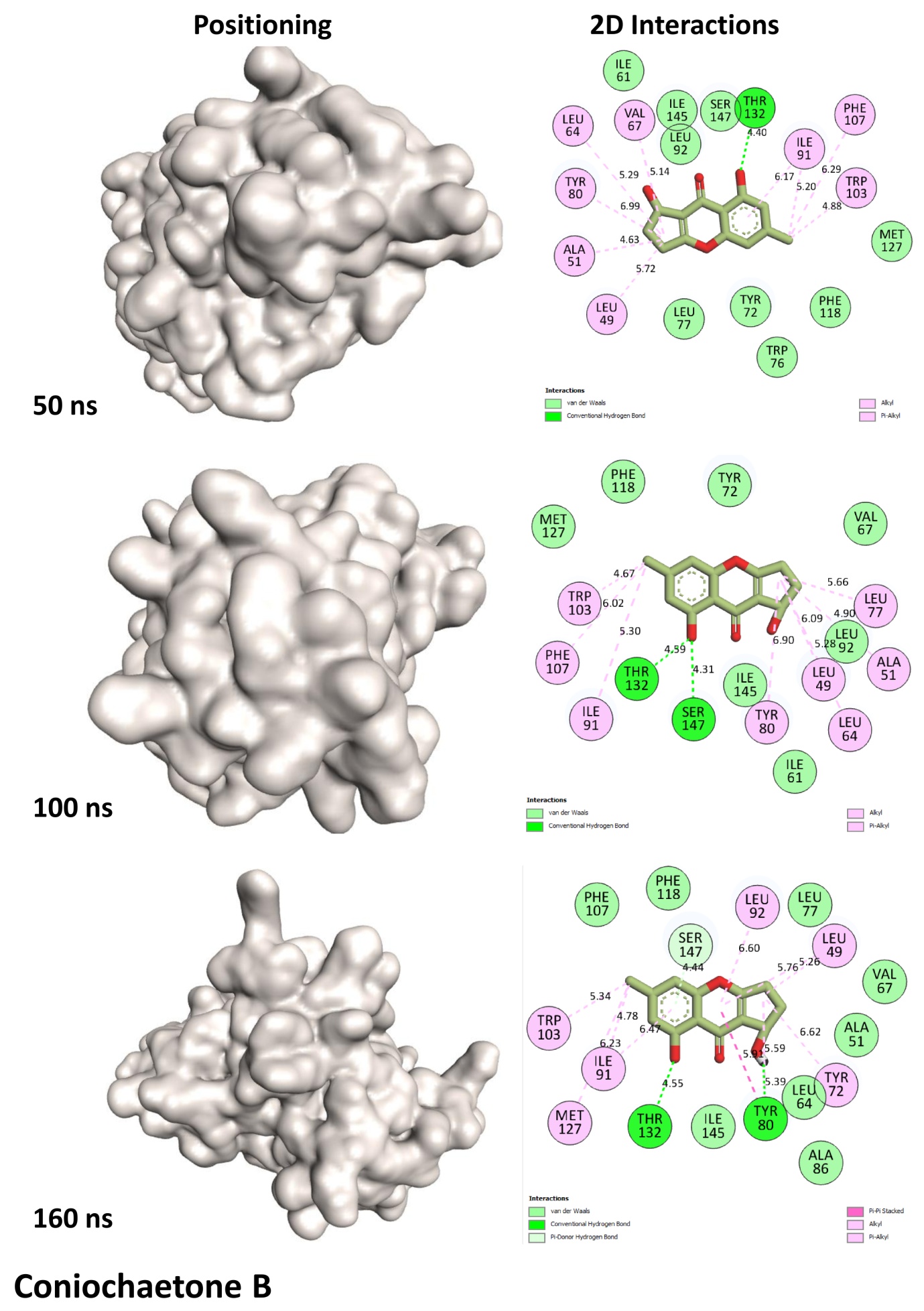
**

**Figure S2.** Interaction plots of Coniochaetone B at different timeframe with CviR during the 160 ns simulation.


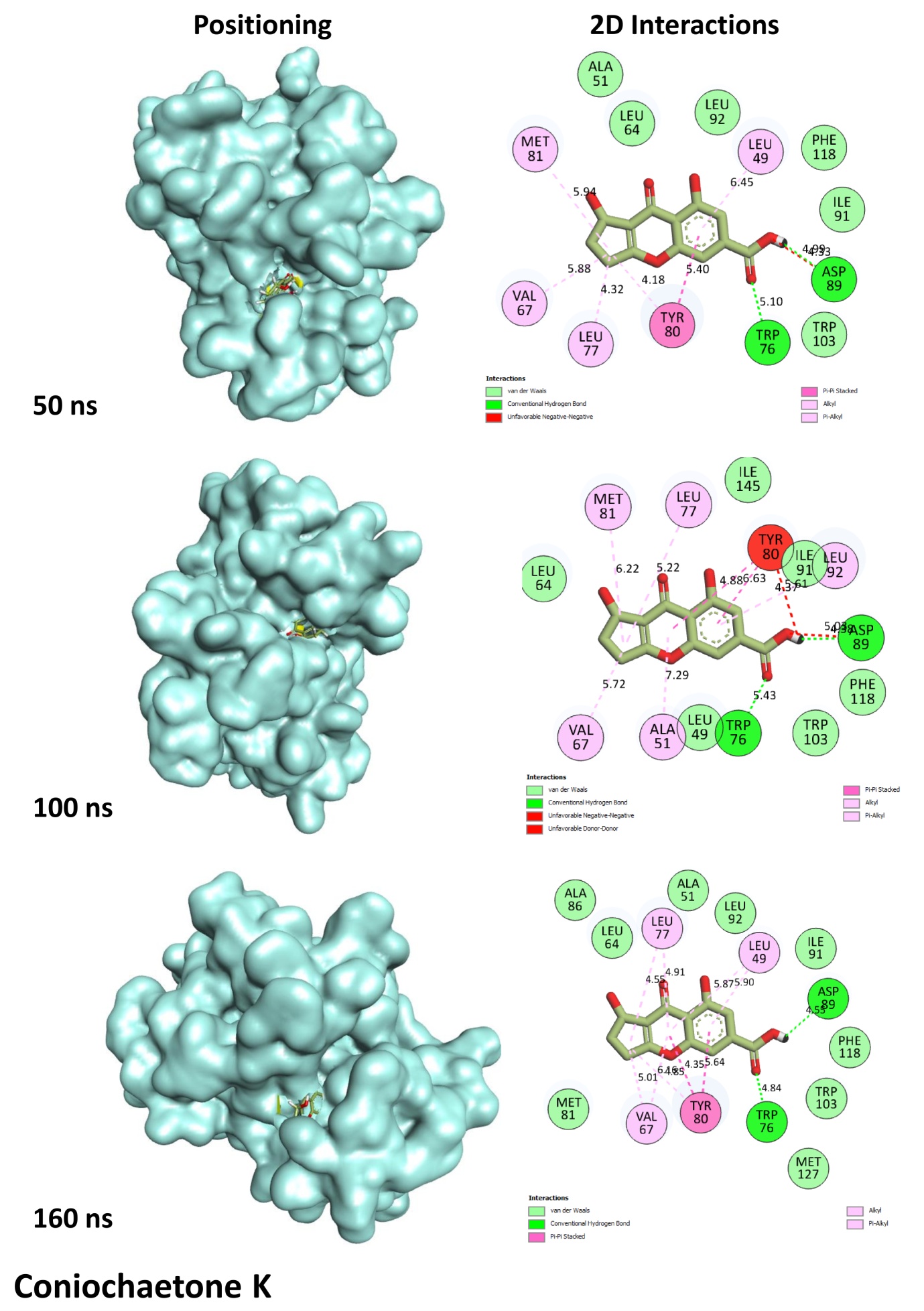


**Figure S3.** Interaction plots of Coniochaetone K at different timeframe with CviR during the 160 ns simulation.


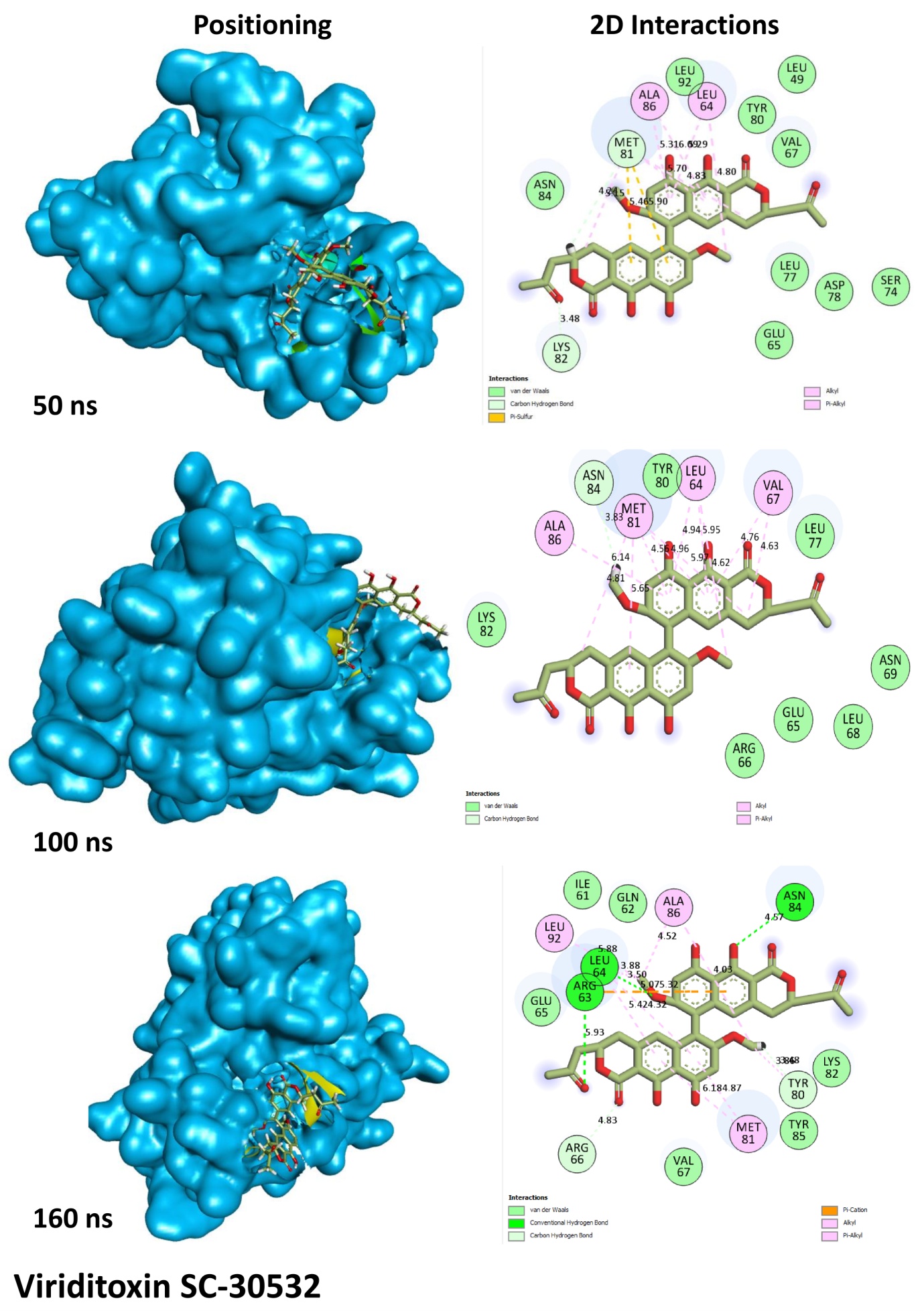


**Figure S4.** Interaction plots of Viriditoxin SC-30532 at different timeframe with CviR during the 160 ns simulation.


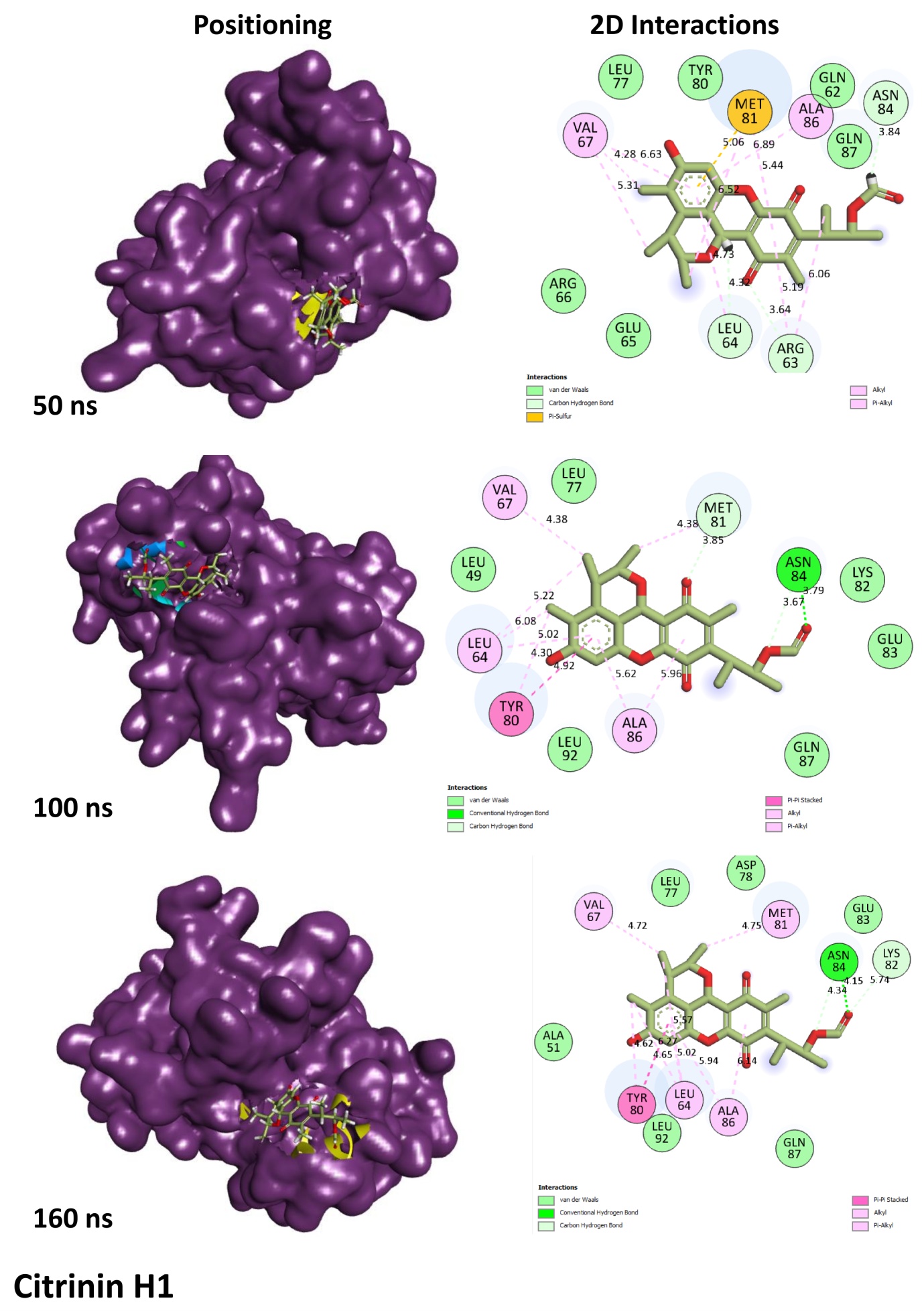


**Figure S5.** Interaction plots of Citrinin H1 at different timeframe with CviR during the 160 ns simulation.


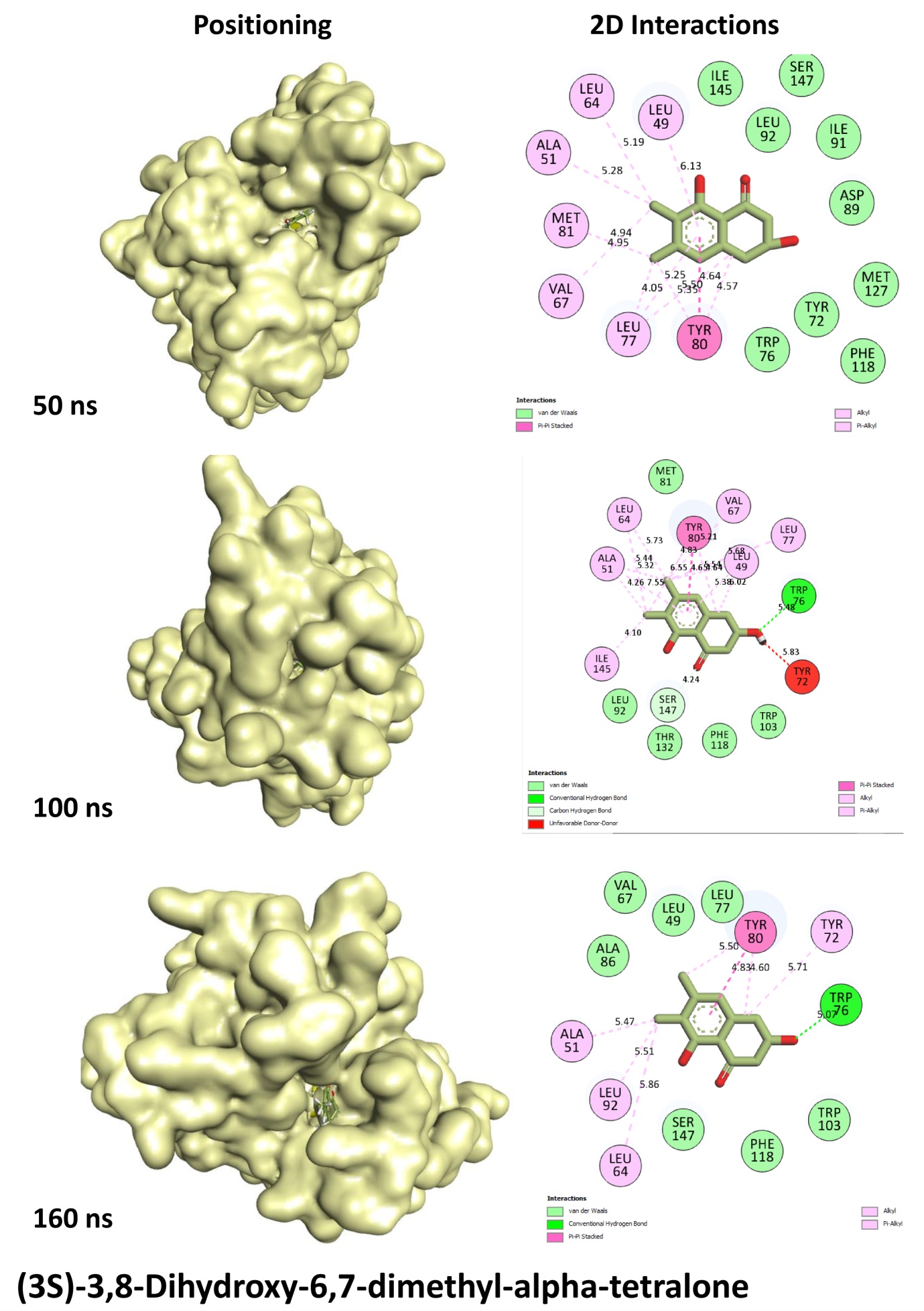


**Figure S6.** Interaction plots of (3S)-3,8-Dihydroxy-6,7-dimethyl-alpha-tetralone at different timeframe with CviR during the 160 ns simulation.

**
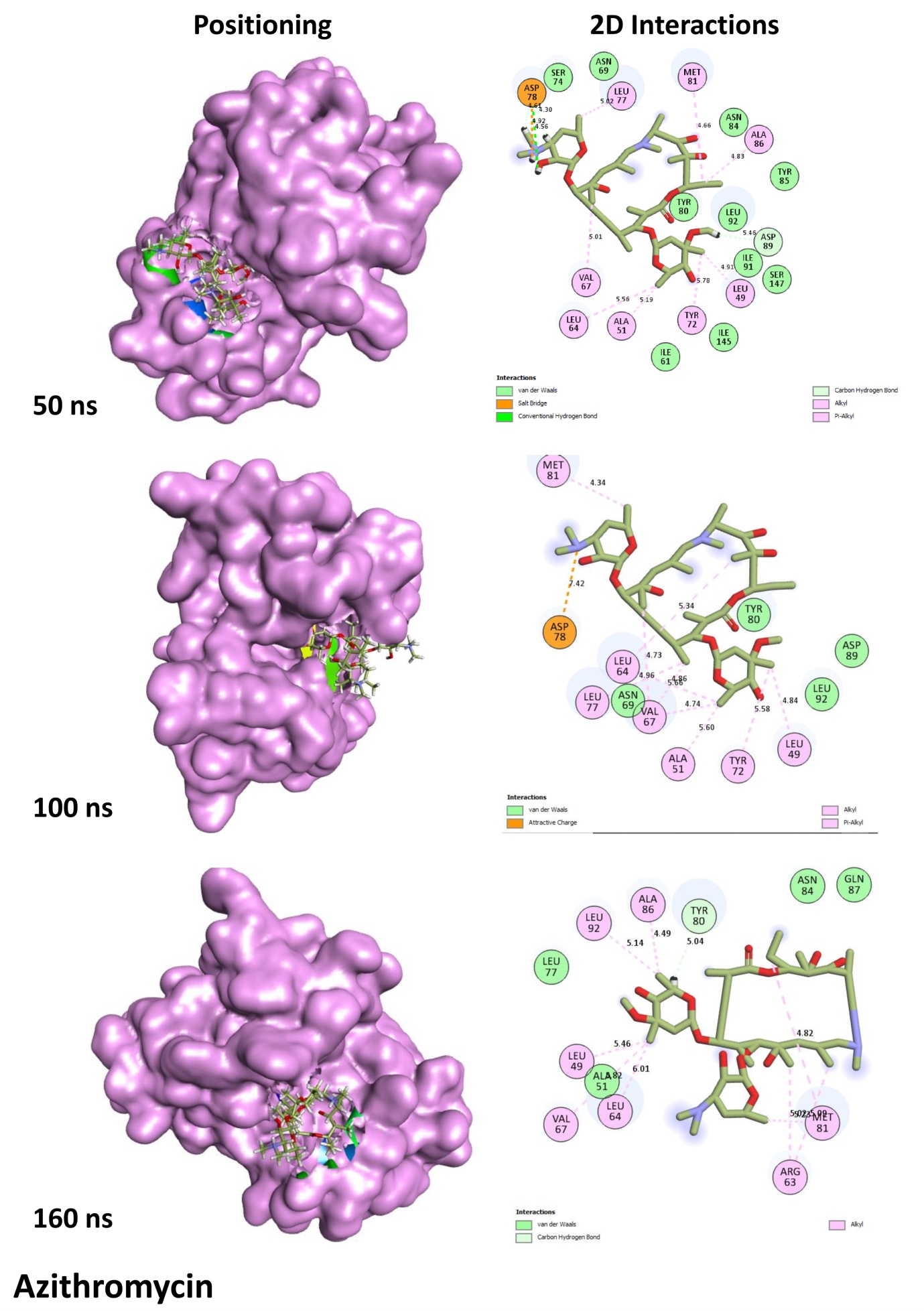
**

**Figure S7.** Interaction plots of Azithromycin at different timeframe with CviR during the 160 ns simulation.
